# Central amygdala micro-circuits mediate fear extinction

**DOI:** 10.1038/s41467-021-24068-x

**Published:** 2021-07-06

**Authors:** Nigel Whittle, Jonathan Fadok, Kathryn P. MacPherson, Robin Nguyen, Paolo Botta, Steffen B. E. Wolff, Christian Müller, Cyril Herry, Philip Tovote, Andrew Holmes, Nicolas Singewald, Andreas Lüthi, Stéphane Ciocchi

**Affiliations:** 1 Friedrich Miescher Institute for Biomedical Research, Basel, Switzerland; 2 Department of Pharmacology and Toxicology, Institute of Pharmacy and CMBI, University of Innsbruck, Innsbruck, Austria; 3 Laboratory of Behavioral and Genomic Neuroscience, National Institute on Alcohol Abuse and Alcoholism, NIH, Bethesda, MD USA; 4 Laboratory of Systems Neuroscience, Department of Physiology, University of Bern, Bern, Switzerland; 5 University of Basel, Basel, Switzerland; 6 Present Address: Department of Psychology and Tulane Brain Institute, Tulane University, New Orleans, LA USA; 7 Present Address: Zuckerman Institute, Columbia University, New York, NY USA; 8 Present Address: Department of Pharmacology, University of Maryland School of Medicine, Baltimore, MD USA; 9 Present Address: INSERM, Neurocentre Magendie, U1215, Bordeaux, France; 10 Present Address: Institute of Clinical Neurobiology, University Hospital Würzburg, Würzburg, Germany

**Keywords:** Amygdala, Neural circuits

## Abstract

Fear extinction is an adaptive process whereby defensive responses are attenuated following repeated experience of prior fear-related stimuli without harm. The formation of extinction memories involves interactions between various corticolimbic structures, resulting in reduced central amygdala (CEA) output. Recent studies show, however, the CEA is not merely an output relay of fear responses but contains multiple neuronal subpopulations that interact to calibrate levels of fear responding. Here, by integrating behavioural, in vivo electrophysiological, anatomical and optogenetic approaches in mice we demonstrate that fear extinction produces reversible, stimulus- and context-specific changes in neuronal responses to conditioned stimuli in functionally and genetically defined cell types in the lateral (CEl) and medial (CEm) CEA. Moreover, we show these alterations are absent when extinction is deficient and that selective silencing of protein kinase C delta-expressing (PKCδ) CEl neurons impairs fear extinction. Our findings identify CEA inhibitory microcircuits that act as critical elements within the brain networks mediating fear extinction.

## Abstract (web summary)

The central amygdala inhibitory microcircuits mediate fear extinction by reversible, stimulus- and context-specific changes in neuronal responses. These alterations are absent when extinction is deficient and selective silencing of PKCδ neurons impairs fear extinction.

## Introduction

The survival of animals depends on their ability to mobilize appropriate defensive behaviours to imminent threats^1^. Yet, animals also need to be able to flexibly adapt to changes in threat contingencies by inhibiting fear responses when threat-related stimuli no longer associate with aversive outcomes, in a process called fear extinction^2–4^. In a typical experimental procedure, an association between conditioned stimuli (CS, a tone or light) and unconditioned stimuli (US, e.g. a foot shock) is first formed (fear conditioning) and then subsequently updated by repeated presentations of the CS in the absence of the US (fear extinction). Fear extinction is thought of as a new learning process wherein animals learn that the CS is no longer predictive of the US^4^. Thus, fear extinction does not reflect the mere erasure of the conditioned fear memory; indeed fear can be spontaneously recovered after fear extinction, or triggered by exposure to the US or a new context^5–7^.

Current models posit that neural circuits and cell assemblies for fear and extinction memories compete with one another, in a context-dependent manner, to determine the degree of fear responding to the CS^4,8,9^. In recent years, there have been major advances in delineating the neural circuity underlying fear conditioning and extinction^10,11^, yet key elements of this circuitry remain to be elucidated. Of particular note, the central nucleus of the amygdala (CEA) has long been ascribed an essential role in the expression of conditioned fear responses^1^. However, detailed dissection of CEA circuitry, using in vivo and ex vivo recordings from functionally and/or genetically defined cell types, has recently challenged the view that the CEA is merely an output relay of fear responses. Instead, this work indicates that the CEA contains multiple anatomically, molecularly and functionally defined neuronal subpopulations that interact to calibrate levels of fear responding^12–14^.

CEA output neurons located in both the lateral (CEl) and the medial (CEm) subdivision of the CEA project to downstream targets that mediate different components of conditioned defensive behaviours, such as freezing or flight^15–17^. Fear conditioning potentiates excitatory input onto CEl projections to the ventrolateral periaqueductal grey (vlPAG)^18^ and CEm output neurons exhibit increased CS responses upon fear conditioning^15^. In turn, the activity of CEm output neurons is thought to be controlled by excitatory glutamatergic afferents from auditory thalamus and the basal amygdala (BA)^19^.

Crucially, however, CEm output neurons are also subject to inhibitory control from GABAergic neurons located in the neighbouring intercalated cell clusters (ITCs) and a subset of neurons in the CEl expressing protein-kinase C delta (PKCδ)^15,20,21^. Thus, following fear conditioning, three functional neuronal subpopulations emerge in the CEl: (1) CS ‘non-responsive’ neurons, (2) CElon neurons that are excited by the CS following fear conditioning and overlap in part with a somatostatin (SST)-expressing population^18,22,23^ and (3) CEloff neurons, which acquire an inhibitory response to the CS and partly overlap with PKCδ neurons^15^. Interestingly, CElon and CEloff neurons can inhibit each other^15^, and SST neurons can inhibit PKCδ neurons^22^, which could result in a switch-like disinhibition of output neurons in CEm or in CEl^12^.

Together, these earlier findings show that there is a layer of processing and plasticity within the CEA that can serve to promote and limit fear responding^24,25^. This raises the intriguing possibility that the same CEA circuitry could be ideally positioned to mediate fear extinction and act as a substrate for the reductions in fear responding that occur with extinction. The major aim of the current study was to test this hypothesis using a combination of behavioural, in vivo electrophysiological, optogenetic and molecular approaches. Our findings demonstrate that microcircuits within the CEA are crucial for fear extinction.

## Results

### Neuronal correlates of fear extinction in subpopulations of CEA neurons

**Fig. 1:**
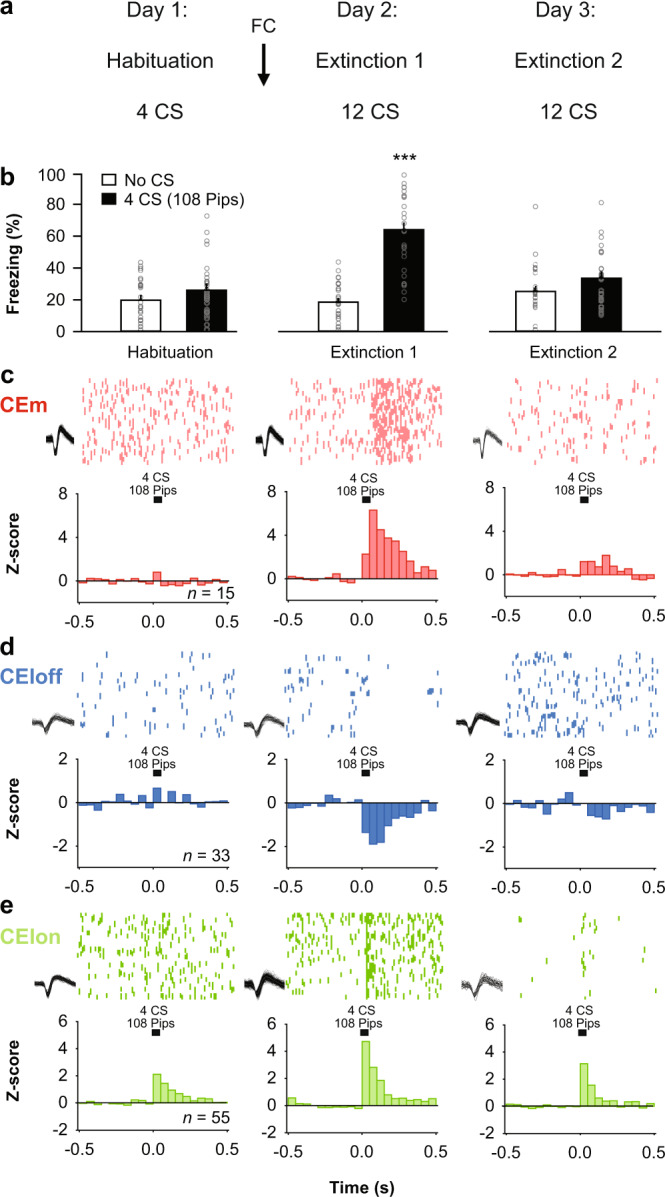
Neuronal correlates of fear extinction in subpopulations of CEA neurons. **a** Behavioural protocol. FC: fear conditioning. CS: conditioned stimuli. **b** Behavioural data. B6 mice: *n* = 27; freezing, habituation, no CS: 19.8 ± 2.6%, CS: 26.1 ± 3.3%, beginning of extinction 1, no CS: 18.5 ± 2.3%, CS: 61.9 ± 4.6%, end of extinction 2, no CS: 25.3 ± 3.1%, CS: 34.1 ± 3.4%, blocks (averages) of 4 CSs. One-way repeated-measures ANOVA *F*_(5,130)_ = 30.8, *p* < 0.001, followed by post hoc Bonferroni *t*-test vs. CS group during habituation, *p* < 0.001. Bar plots are expressed as means ± SEM. Circles are freezing values of individual mice. **c** Raster plots and corresponding spike waveforms of a representative CEm unit (top). Normalized and averaged population peri-stimulus time histograms (bottom). CEm neurons: *n* = 15 units from 5 mice; *z*-score habituation: −0.11 ± 0.45, beginning of extinction 1: 4.21 ± 1.75, end of extinction 2: 1.24 ± 0.48, blocks of 4 CSs. One-way repeated-measures ANOVA *F*_(2,28)_ = 3.9, *p* = 0.033 followed by post hoc Bonferroni *t*-test vs. during habituation, *p* = 0.023. **d** Raster plots and corresponding spike waveforms of a representative CEloff unit (top). Normalized and averaged population peri-stimulus time histograms (bottom). CEloff neurons: *n* = 33 units from 18 mice; *z*-score, habituation: 0.28 ± 0.33, beginning of extinction 1: −1.53 ± 0.28, end of extinction 2: −0.46 ± 0.34, blocks of 4 CSs. One-way repeated-measures ANOVA *F*_(2,64)_ = 8.4, *p* < 0.001 followed by post hoc Bonferroni *t*-test vs. during habituation, *p* < 0.001. **e** Raster plots and corresponding spike waveforms of a representative CElon unit (top). Normalized and averaged population peri-stimulus time histograms (bottom). CElon neurons: *n* = 55 units from 15 mice; *z*-score, habituation: 1.30 ± 0.30, beginning of extinction 1: 2.54 ± 0.43, end of extinction 2: 1.40 ± 0.30, blocks of 4 CSs. One-way repeated-measures ANOVA *F*_(2,108)_ = 5.3, *p* = 0.006 followed by post hoc Bonferroni *t*-test vs. during habituation, *p* = 0.008. All individual neurons of each CEA population had significant *z*-score values upon CS presentation (4 first CSs during extinction 1). Source data are provided as a Source data file.

To first identify neuronal correlates of fear extinction in CEA circuits, we submitted freely-moving mice (*n* = 27, C57BL/6J, hereafter B6) to a fear-conditioning and extinction procedure while chronically recording single-unit activity in CEA (Figs. S1, S2; Supplementary Tables 1–4; see ‘Methods’)^15^. Following fear conditioning, mice exhibited a selective increase in conditioned freezing to the CS that was reversed to pre-conditioning levels by the end of fear extinction learning (Fig. 1a, b; Fig. S3).

Examination of the CS-related activity of CEA single units during testing revealed three subpopulations exhibiting distinct patterns of responding that were reversed from fear conditioning to extinction. Fear-conditioning-induced differential conditioned responses in CEm and CEl neurons, as previously reported^15,17^, such that CEm neurons increased their phasic CS responses (Fig. 1c), while CEl neurons exhibited either an inhibitory response (Fig. 1d) or an increase (Fig. 1e) in conditioned responses, consistent with the activity of CEloff and CElon neurons, respectively (Figs. S4, S5). A larger proportion of CElon neurons (64%, 35 out of 55 neurons) exhibited cue-related responses during habituation, as compared to CEloff (24%, 8 out of 33 neurons) and CEm neurons (27%, 4 out of 15 neurons). Strikingly, fear conditioning-related changes in the CS responses of all three neuronal subpopulations were reversed following fear extinction, i.e., elevated CS-related activity in CEm and CElon neurons was attenuated and the inhibited CS-related activity of CEloff neurons was diminished (Fig. 1c–e). These data suggest that changes in CS-related phasic activity within the CEA inhibitory microcircuits signal the extinction of fear responses.

### CEA subpopulation activity tracks extinction-related changes in the expression of fear

**Fig. 2:**
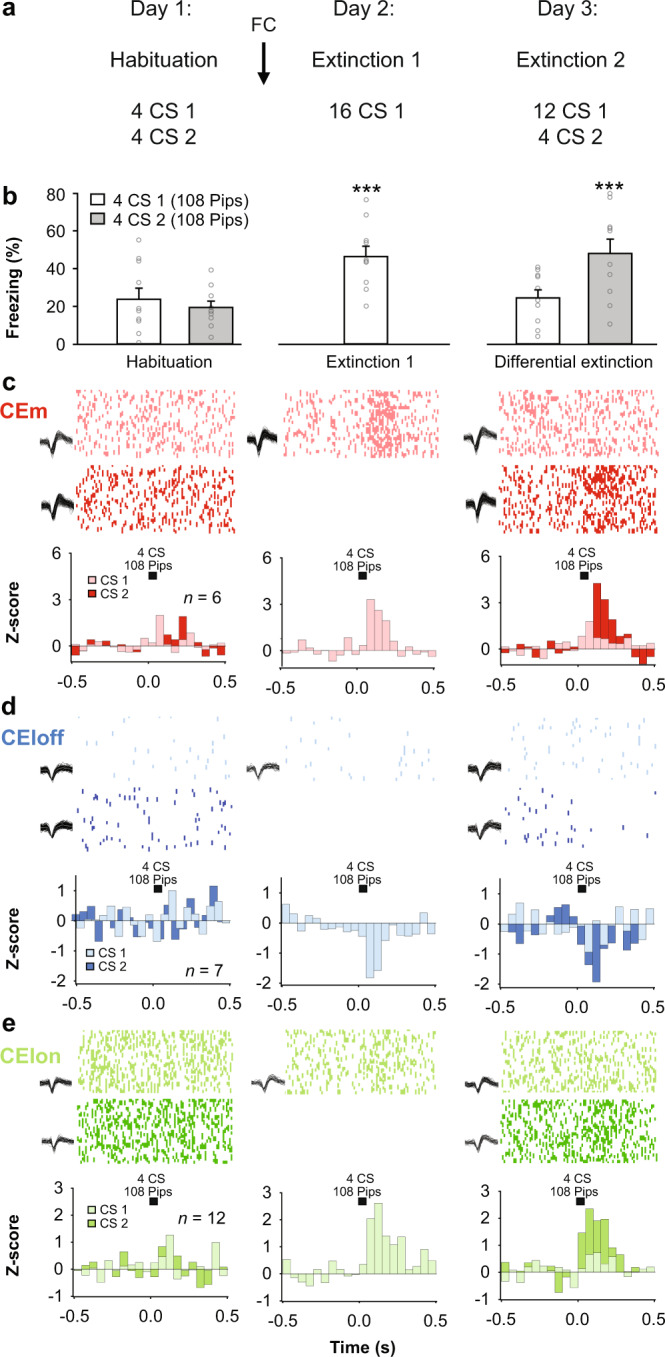
CEA subpopulation activity tracks extinction-related changes in the expression of conditioned freezing. **a** Behavioural protocol. FC: fear conditioning. CS: conditioned stimuli. **b** Freezing behaviour. B6 mice: *n* = 10; freezing, habituation, CS1: 23.8 ± 5.9%, CS2: 19.5.1 ± 3.3%, beginning of diff. extinction 1, CS1: 46.4 ± 5.4%; end of diff. extinction 2: CS1: 24.5 ± 4.3%, CS2: 48.1 ± 7.6%, blocks of 4 CSs. One-way repeated-measures ANOVA *F*_(4,36)_ = 8.6, *p* < 0.001, followed by post hoc Bonferroni *t*-test vs. CS2 block during habituation, *p* < 0.001. Bar plots are expressed as means ± SEM. Circles are freezing values of individual mice. **c** Raster plots and corresponding spike waveforms of a representative CEm unit (top). Normalized and averaged population peri-stimulus time histograms (bottom). CEm neurons: *n* = 6 units from 3 mice; *z*-score, habituation, CS1: −0.21 ± 0.44, CS2: 0.64 ± 0.38, beginning of diff. extinction 1, CS1: 2.66 ± 0.87; end of diff. extinction 2: CS1: 0.70 ± 0.42, CS2: 3.79 ± 1.08, blocks of 4 CSs. One-way repeated-measures ANOVA *F*_(4,20)_ = 5.2, *p* = 0.005 followed by post hoc Bonferroni *t*-test vs. CS1 block during habituation, *p* < 0.05. **d** Raster plots and corresponding spike waveforms of a representative CEloff unit (top). Normalized and averaged population peri-stimulus time histograms (bottom). CEloff neurons: *n* = 7 units from 5 mice; *z*-score, habituation, CS1: 0.32 ± 0.26, CS2: −0.24 ± 0.29, beginning of diff. extinction 1, CS1: −1.33 ± 0.47; end of diff. extinction 2: CS1: −0.51 ± 0.72, CS2: −1.37 ± 0.35, blocks of 4 CSs. One-way repeated-measures ANOVA *F*_(4,24)_ = 3.4, *p* = 0.023 followed by post hoc Bonferroni *t*-test vs. CS1 block during habi*t*uation, *p* < 0.05. **e** Raster plots and corresponding spike waveforms of a representative CElon unit (top). Normalized and averaged population peri-stimulus time histograms (bottom). CElon neurons: *n* = 12 units from 5 mice; *z*-score, habituatio*n*: CS1: −0.40 ± 0.21, CS2: 0.41 ± 0.23 beginning of diff. extinction 1: CS1: 1.60 ± 0.39; end of diff. extinction 2: CS1: 0.46 ± 0.27, CS2: 1.92 ± 0.53, blocks of 4 CSs. One-way repeated-measures ANOVA *F*_(4,44)_ = 6.0, *p* < 0.001 followed by post hoc Bonferroni *t*-test vs. CS1 block during habituation, *p* < 0.05. All individual neurons of each CEA population had significant *z-*score values upon CS presentation (first 4 CSs during diff. extinction 1). Source data are provided as a Source data file.

How specific is the reversal of CEA subpopulation activity during fear extinction? Does it correlate with behavioural changes and emotional values of conditioned stimuli, or does it simply reflect a non-associative process such as stimulus habituation or desensitisation? To test for the selectivity of fear extinction-induced reversal of neuronal responses in CEA microcircuits, we trained mice (*n* = 10) using a discriminative fear extinction paradigm (Fig. 2a). In this task, mice were conditioned to two different CSs followed by the extinction of just one of these CSs. Immediately after the extinction, animals were exposed to the non-extinguished CS—which resulted in an instantaneous switch (low to high) in fear behaviour (Fig. 2b).

We reasoned that if the neural responses in CEA subpopulations correlate with fear extinction per se, rather than with non-associative or time-related processes, we should expect a change in neural activity paralleling the behavioural switch from the extinguished to the non-extinguished CS. In line with this prediction, we observed that the switch in behaviour corresponded to an immediate recovery of CS-induced responses in CEm, CEloff and CElon neurons to levels of activity evident after fear conditioning (Fig. 2c–e). Thus, CS-related responses in these CEA subpopulations track extinction-induced changes in the expression of fear responses to the CS.

### Context-driven fear renewal reverses extinction-related CEA subpopulation activity

**Fig. 3:**
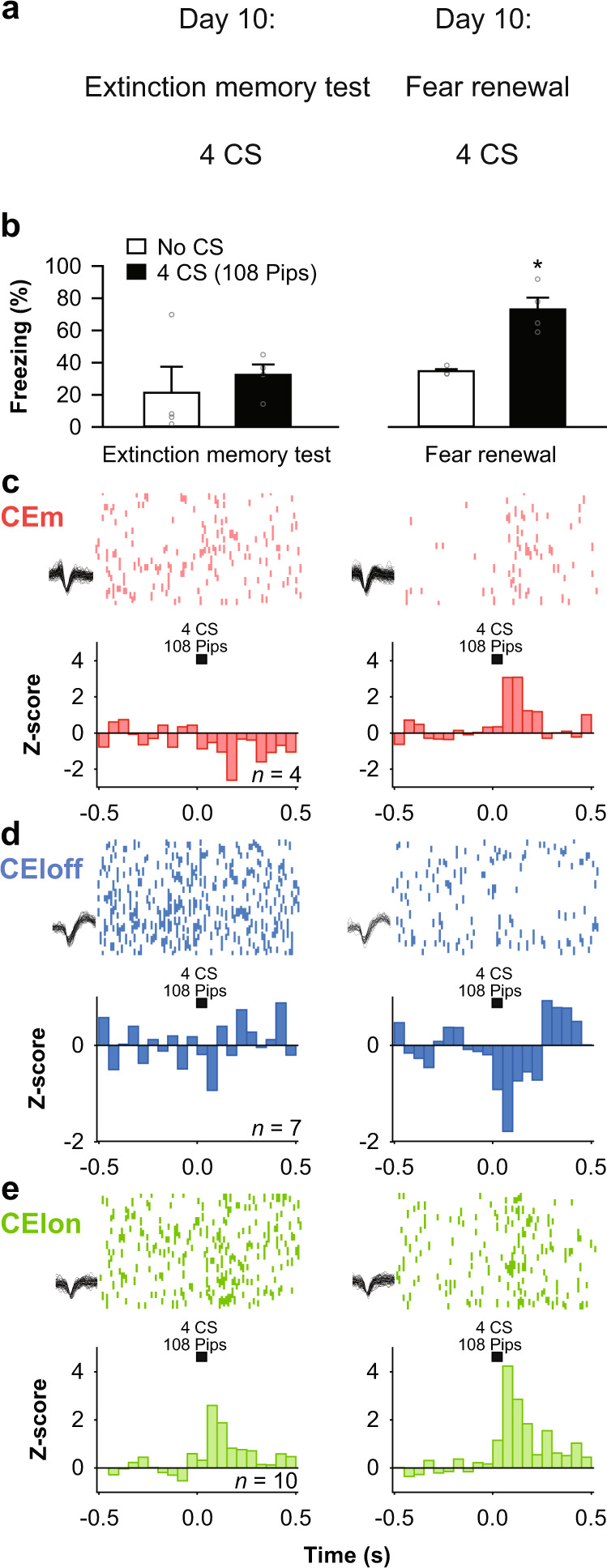
Context-driven fear renewal reverses extinction-related CEA subpopulation activity. **a** Behavioural protocol. CS: conditioned stimuli. **b** Behavioural data. B6 mice: *n* = 4; extinction memory retrieval, no CS: 21.4 ± 16.2%, CS: 32.5 ± 6.4%, fear renewal, no CS: 34.8 ± 1.2%, CS: 73.0 ± 7.3%, blocks of 4 CSs. One-way repeated-measures ANOVA *F*_(3,9)_ = 8.5, *p* = 0.005 followed by post hoc Bonferroni *t*-test vs. CS block during extinction memory, *p* = 0.015. Bar plots are expressed as means ± SEM. Circles are freezing values of individual mice. **c** Raster plots and corresponding spike waveforms of a representative CEm unit (top). Normalized and averaged population peri-stimulus time histograms (bottom). CEm neurons: *n* = 4 units from 1 mouse; *z*-score, extinction memory retrieval: −0.79 ± 1.59, fear renewal: 3.08 ± 1.06, blocks of 4 CSs. Paired student *t-*test, two-sided, *p* = 0.039. **d** Raster plots and corresponding spike waveforms of a representative CEloff unit (top). Normalized and averaged population peri-stimulus time histograms (bottom). CEloff neurons: *n* = 7 units from 2 mice; *z*-score, extinction memory retrieval: −0.04 ± 0.61, fear renewal: −0.94 ± 0.40, blocks of 4 CSs. Paired student *t-*test, two-sided, *p* = 0.041. **e** Raster plots and corresponding spike waveforms of a representative CElon unit (top). Normalized and averaged population peri-stimulus time histograms (bottom). CElon neurons: *n* = 10 units from 2 mice; *z*-score, extinction memory: 1.28 ± 0.41, fear renewal: 2.13 ± 0.37, blocks of 4 CSs. Paired student *t-*test, two-sided, *p* = 0.034. All individual neurons of each CEA population had significant *z*-score values upon CS presentation (4 CSs during fear renewal). Source data are provided as a Source data file.

Fear extinction does not simply reflect forgetting of the CS-US associations, but a new learning process subjected to contextual modulation^4,26^. This sensitivity to the context provided us with an opportunity to test whether changes in CS-related activity in CEA subpopulations is sensitive to contextual changes. One week following fear extinction learning, memory for the extinguished CS was observed in a subset of mice (Fig. 3a, b): these mice expressed low levels of CS-induced freezing when tested in the context in which extinction learning occurred. However, when these same mice were tested in the conditioning context, there was an expected ‘renewal’ of CS-induced conditioned freezing (Fig. 3b, Supplementary Table 5).

We asked whether this context-induced reversion of fear responding was paralleled by an alteration in CEA single-unit activity. Indeed, while all three CEA neuronal populations showed reduced CS responses during the retrieval of extinction memory in the extinction context (Fig. 3c–e; Fig. S6), CS-related responses were completely re-established to pre-extinction levels when testing occurred in the conditioning context (Fig. 3c–e). Thus, the context-gated expression of extinguished fear memory closely parallels changes in the activity of the CEA subpopulations.

### Impaired extinction associates with persistent fear-related activity in CEA subpopulations

**Fig. 4:**
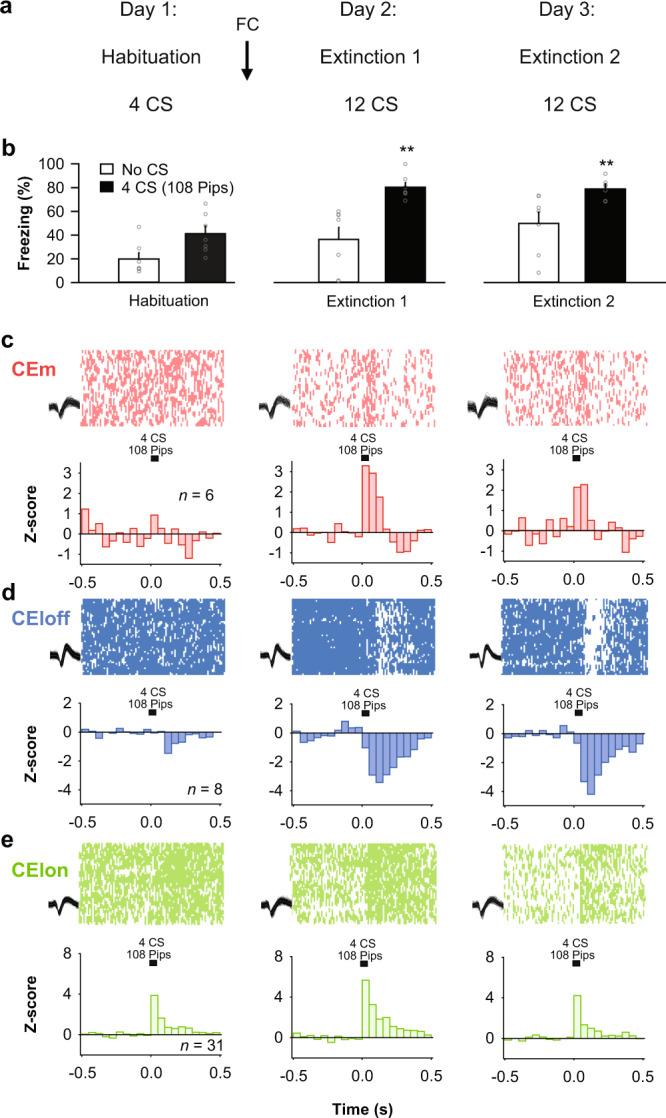
Impaired extinction correlates with persistent fear-related activity in CEA subpopulations. **a** Behavioural protocol. FC: fear conditioning. CS: conditioned stimuli. **b** Behavioural data. S1 mice: *n* = 7; habituation, no CS: 19.9 ± 5.1%, CS: 41.2 ± 6.3%, beginning of extinction 1, no CS: 36.4 ± 10.1%, CS: 80.3 ± 3.8%, end of extinction 2, no CS: 49.8 ± 9.5%, CS: 78.9 ± 3.2%, blocks of 4 CSs. One-way repeated-measures ANOVA *F*_(5,30)_ = 12.0, *p* < 0.001, followed by post hoc Bonferroni *t*-test vs. CS block during habituation, *p* < 0.01. Bar plots are expressed as means ± SEM. Circles are freezing values of individual mice. **c** Raster plots and corresponding spike waveforms of a representative CEm unit (top). Normalized and averaged population peri-stimulus time histograms (bottom). CEm neurons: *n* = 6 units from 2 mice; *z*-score, habituation: 0.45 ± 0.42, beginning of extinction 1: 3.12 ± 0.73, end of extinction 2: 2.21 ± 0.48, blocks of 4 CSs. One-way repeated-measures ANOVA *F*_(2,10)_ = 5.9, *p* = 0.020 followed by post hoc Bonferroni *t*-test vs. during habituation, *p* < 0.05. **d** Raster plots and corresponding spike waveforms of a representative CEloff unit (top). Normalized and averaged population peri-stimulus time histograms (bottom). CEloff neurons: *n* = 8 units from 6 mice; *z*-score, habituation: −0.49 ± 0.87, beginning of extinction 1: −2.46 ± 1.13, end of extinction 2: −2.73 ± 1.87, blocks of 4 CSs. One-way repeated-measures ANOVA *F*_(2,14)_ = 4.2, *p* = 0.037 followed by post hoc Bonferroni *t*-test vs. during habituation, *p* < 0.05. **e** Raster plots and corresponding spike waveforms of a representative CEloff unit (top). Normalized and averaged population peri-stimulus time histograms (bottom). CElon neurons: *n* = 31 units from 6 mice; *z-*score, habituation: 1.60 ± 0.38, beginning of extinction 1: 3.09 ± 0.48, end of extinction 2: 1.82 ± 0.46, blocks of 4 CSs. One-way repeated-measures ANOVA *F*_(2,60)_ = 3.8, *p* = 0.028 followed by post hoc Bonferroni *t*-test vs. during habituation, *p* < 0.05. All individual neurons of each CEA population had significant *z*-score values upon CS presentation (first 4 CSs during extinction 1). Source data are provided as a Source data file.

Impaired fear extinction is a hallmark of anxiety disorders^27^, yet endogenous neural correlates of impaired fear extinction in CEA have not been thoroughly investigated in vivo. Based on our findings thus far, we reasoned that impaired extinction would correspond with the persistence of a ‘fear-like’ pattern of activity in CEA subpopulations. To test this prediction, we performed CEA single-unit recordings in a mouse strain (129S1/SvImJ, ‘S1’) that exhibits impaired extinction and associated abnormalities in CEA IEG activity^28^. We first replicated prior data^28–30^ showing that S1 mice tested using the same procedures used in earlier experiments in the current study, failed to exhibit a reduction in CS-related freezing after extinction training (*n* = 7 mice, Fig. 4a, b; Fig. S3).

Examination of the CEA single-unit activity in the S1 mice revealed activity patterns in the same three CEA neuronal subpopulations as described above (CElon, CEloff and CEm) (Fig. 1). Strikingly, however, and entirely in keeping with the absence of extinction at the behavioural level, the patterns of CEA activity evident after fear conditioning were largely unchanged after extinction (Fig. 4c–e). Specifically, CEm neurons showed a persistent increase in their phasic CS responses after fear conditioning and extinction, and the inhibitory response of CEloff neurons that emerged after conditioning was also maintained after extinction. Interestingly, however, the conditioning-related increase in the CS-related activity CElon neurons was partially restored to pre-conditioning levels after extinction. This is notable because it shows that CS-related inhibition of CEloff neurons does not require input from CElon neurons and that, by extension, other upstream populations (e.g., ITC neurons) can drive this inhibition.

These data suggest that plastic changes in the activity of CEloff neurons may be a pivotal step in the reduction in fear responses produced by fear extinction, and that the failure of this plasticity may underlie the extinction deficits evident in S1 mice. To further test this hypothesis, we took advantage of the fact that PKCδ CEl neurons overlap with CEloff neurons^21^ to compare, via immunostaining, the expression of the immediate-early gene (IEG) Zif268 in immunolabeled PKCδ CEl neurons of S1 and B6 mice after either a fear retrieval or extinction learning test (Fig. S7a). We found that the number of neurons positive for both Zif268 and PKCδ in CEl was increased in extinguished B6 mice, relative to non-extinguished fear-tested counterparts. By contrast, S1 mice did not show an extinction-related increase in Zif268/PKCδ neurons, consistent with a failure to reverse CS-related inhibition of the CEloff subpopulation (Fig. S7a). This was not due to a lower number of PKCδ neurons in CEl of S1 mice, as the overall number of these neurons was similar in the two strains (Fig. S7a). Detailed analysis of the dendritic morphology of CEA neurons in test-naive mice did, however, indicate evidence of more overall dendritic material in CEA neurons of S1, relative to B6 mice, indicating plasticity deficits in CEloff neurons of S1 mice may relate to underlying structural abnormalities (Fig. S7b).

### Activity of PKCδ/CEloff neurons during CS exposure is required for extinction memory formation

Collectively, our findings thus far suggest that extinction-associated neuronal plasticity in CEA circuits may be necessary for the successful acquisition and expression of extinction memories. In particular, our data posit a critical circuit mechanism in which CEloff neurons gate reductions in fear seen with extinction by exerting inhibition of CEm neurons. Alternatively, changes in the activity in these CEA subpopulations during fear extinction could simply reflect the relaying of upstream plasticity mechanisms [e.g., from basolateral amygdala (BLA) or medial prefrontal cortex] to downstream targets (e.g., vlPAG). Furthermore, though a prior study found that inactivating CEloff neurons throughout fear conditioning and retrieval increased freezing responses^21^, it remains unclear whether CEloff neurons causally contribute to the decrease in freezing that occurs with extinction.

**Fig. 5:**
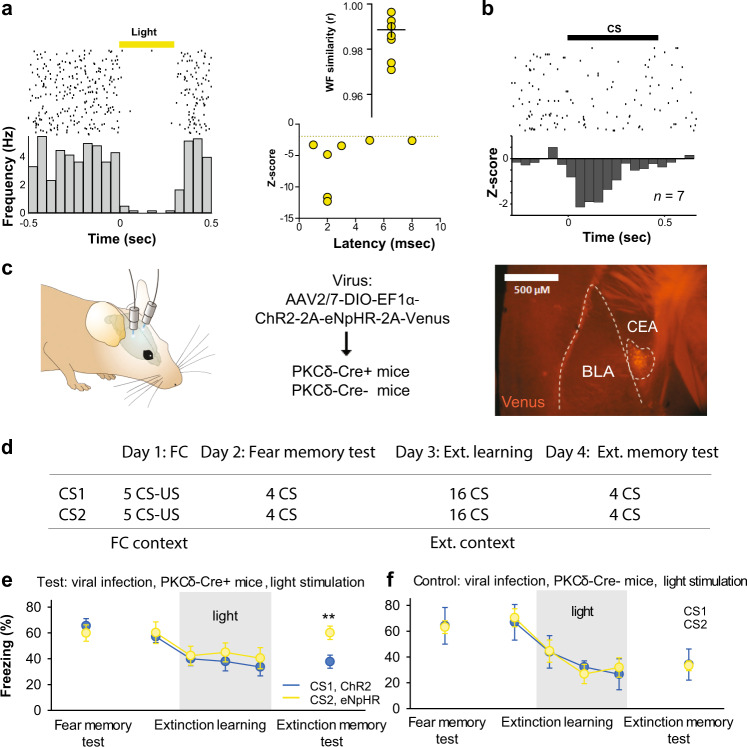
PKCδ/CEloff neuronal activity during CS exposure is required for extinction memory formation. **a** Left, PKCδ unit identified with optogenetics. Right, waveform similarity of spikes with or without optogenetic stimulation. Bottom right, latency and magnitude of inhibition of PKCδ/CEloff neurons. **b** Normalized activity (*z*-score, bottom) of PKCδ/CEloff cells (*n* = 7) and example raster plot (top). CS: conditioned stimuli. **c** An adeno-associated virus (AAV2/7) conditionally expressing ChR2, eNpHR and a Venus reporter under the control of an elongation factor-1α (EF-1α) promoter was injected into the CEl of a PKCδ-Cre+ or PKCδ-Cre− mice (middle). Anti-GFP immunolabelling of the Venus reporter gene in CEl (right). DIO: double-inverted open reading frame; PKCδ: protein-kinase c delta; 2A: ribosomal self-processing peptide; BLA: basolateral amygdala; CEA: central amygdala. **d** Behavioural protocol. **e** Freezing response in PKCδ-Cre+ mice expressing ChR2 and eNpHR in PKCδ neurons. B6 mice: *n* = 11; freezing, ChR2, fear memory, CS I: 65.5 ± 5.6%, extinction learning, CS I: 57.1 ± 4.5%, CS II: 39.9 ± 5.2%, CS III: 37.9 ± 7.3%, CS IV: 33.7 ± 7.0%, extinction memory, CS I: 37.7 ± 5.0. eNpHR, fear memory, CS I: 59.9 ± 6.3%, extinction learning, CS I: 60.1 ± 8.4%, CS II: 42.4 ± 7.3%, CS III: 44.9 ± 7.4%, CS IV: 40.5 ± 8.2%, extinction memory, CS I: 60.1 ± 5.2% (blocks of 4 CSs). Main effect of CS presentations during extinction learning: two-way repeated-measures ANOVA *F*_(5,49)_ = 8.7, *p* < 0.001. Interaction of light stimulation and CS presentations: two-way repeated-measures ANOVA *F*_(5,49)_ = 2.34, *p* = 0.056 followed by post hoc Bonferroni *t*-test vs. CS1 block during fear memory, *p* < 0.01. All values are expressed as means ± SEM. **f** Control experiment. Freezing response in PKCδ-Cre− mice not expressing ChR2 and eNpHR in PKCδ neurons. B6 mice: *n* = 5; freezing, blue light, fear memory, CS I: 63.1 ± 4.9%, extinction learning, CS I: 70.4 ± 9.7%, CS II: 44.6 ± 15.0%, CS III: 26.8 ± 8.2%, CS IV: 31.8 ± 13.5%, extinction memory, CS I: 33.2 ± 3.8%. Yellow light, fear memory, CS I: 64.1 ± 14.2%, extinction learning, CS I: 66.9 ± 12.2%, CS II: 43.9 ± 6.1%, CS III: 32.2 ± 4.1%, CS IV: 26.6 ± 5.7%, extinction memory, CS I: 34.1 ± 12.1% (blocks of 4 CSs). Main effect of CS presentations during extinction: two-way repeated-measures ANOVA *F*_(5,20)_ = 15.5, *p* < 0.001. Interaction of light stimulation and CS presentations: two-way repeated-measures ANOVA *F*_(5,20)_ = 0.12, *p* = 0.987 followed by post hoc Bonferroni *t*-test vs. CS1 block during fear memory, *p* > 0.05 for all comparisons. All values are expressed as means ± SEM. Source data are provided as a Source data file.

To address these questions, we took advantage of the fact that PKCδ CEl neurons overlap with CEloff neurons^21^ by using PKCδ::Cre mice (*n* = 5) to selectively express, in a Cre-dependent manner, the inhibitory opsin, Arch (AAV5-DIO-Arch), in PKCδ CEA neurons. We then performed optogenetic phototagging experiments to confirm selective control over the activity of these neurons by shining yellow light into the CEA and showing that this reduced the activity of a subset of single units identifiable as PKCδ neurons (Fig. 5a, b; see ‘Methods’). Furthermore, we examined the activity of these photo-identified neurons after fear conditioning and confirmed that the majority (7/12) exhibited an inhibitory response to the CS, consistent with their designation as CEloff neurons (Fig. 5b, Fig. S8).

Next, we tested whether activating or inhibiting the activity of CEl PKCδ neurons during extinction learning affected freezing responses. To do so, we bilaterally transfected CEl PKCδ neurons in PKCδ::Cre mice (*n* = 11) with an AAV conditionally expressing both the excitatory opsin, channelrhodopin-2 (ChR2), and the inhibitory opsin, halorhodopsin (eNpHR) (Fig. 5c). Mice were then equivalently fear-conditioned to two CSs (CS1 and CS2), as evidenced by similar freezing responses to both CSs during a fear retrieval test (Fig. 5e, f). On a subsequent extinction training session, during each of the last 12 CS1 presentations, blue light was shone to excite PKCδ neurons for 300 ms from CS onset—thereby matching the duration of CS-related activity we had observed in the CEloff neurons (see Fig. 1d, Fig. S9). This was followed by 16 presentations of the CS2, during which yellow light was shone over each of the last CS2 to inhibit these same PKCδ neurons.

We found that freezing responses did not differ between the CS1/ChR2 and CS2/eNpHR groups during extinction learning, indicating that manipulating the activity of this subpopulation during a specific temporal window corresponding to CS presentation does not produce acute changes in freezing responses (Fig. 5e). However, when we examined performance during a subsequent extinction memory test, conducted in the absence of light, we found that freezing levels were higher in the CS2/eNpHR than the CS1/ChR2 group (Fig. 5e). In fact, the freezing levels in the CS2/eNpHR group were similar to the levels seen prior to extinction learning, consistent with a failure to form a lasting extinction memory to CS2 when PKCδ/CEloff neurons were inhibited during extinction training. Importantly, when we repeated the same procedures in PKCδ::Cre-negative mice, freezing did not differ between groups at any stage of testing, excluding technical artefacts (Fig. 5f, Fig. S10). Together, these data show that activity of PKCδ/CEloff neurons during CS exposure is required for the formation of fear extinction memories.

## Discussion

While the CEA is known to play an essential role in the formation and expression of conditioned fear memories, the precise nature of this role is still uncertain^10,11^. Recent studies have shown that fear conditioning potentiates inputs from the lateral amygdala (LA) and paraventricular nucleus of the thalamus (PVT) onto SST-expressing neurons in CEl subdivision of the CEA^14,18^, while inactivation of entire CEl causes fear learning deficits^15^. Furthermore, optogenetically-guided electrophysiological recordings (‘phototagging’) have demonstrated that SST-expressing CEl neurons correspond to a functional class of ‘CElon’ neurons^18^, which induce freezing to a CS^13^ via direct projections to vlPAG^23^ or, alternatively, by gating CEm output through disinhibition mediated by PKCδ (‘CEloff’) neurons located in CEl^15,21^.

In the current study, we found that CElon neurons exhibited significant cue-related responses during habituation, while these were much less apparent for CEloff and CEm neurons. Hence, CElon neurons might encode an attentional or salience-related signal that could reflect preferential innervation by upstream sensory and attention processing regions. Following fear conditioning, a larger increase in CS-related activity in CElon neurons (e.g., through synaptic plasticity occurring during fear learning at afferent synapses to CElon neurons) may then be transmitted locally to CEloff neurons, and ultimately disinhibit CEm output neurons to elicit freezing responses.

Importantly, we found that the CS responses of CElon neurons were reduced by fear extinction, although some level of CS-related activity, similar to that seen during habituation, remained. This extinction-related reduction was also seen in CElon neurons in mice from the S1 mouse strain, despite these animals showing persistent fear. This implies that persistent fear-related activity in CEloff/CEm neurons is mediated by mechanisms separate from CElon neurons, such as changes in input from neighbouring ITCs. Altogether, these findings provide further evidence that the CEA contains a functionally diverse set of neuronal subpopulations coding for responses to conditioned fear stimulus.

Our findings also demonstrate that stimulus-related responses in these neuronal populations are dynamically modified following extinction to calibrate appropriate levels of freezing. Using a combination of in vivo single-unit recordings and optogenetics, we show that extinction-related reductions in freezing correspond to a relative increase in the CS-related activity of fear-inhibiting CEloff neurons and a decrease in the activity of fear-inducing CElon and CEm neurons. Importantly, we found that these changes in CS-related activity are rapidly reversed when fear responding is renewed by exposure to the fear conditioning context and fail to develop to a non-extinguished CS or in S1 mice that show persistent fear responding due to impaired extinction. Thus, the activity of these subpopulations of CEA neurons closely tracks shifts in the emotional significance of the CS that occurs with extinction, and is not simply due to non-associative processes, such as habituation or desensitization, that can occur with repeated CS exposure or the passage of time^4,31^. Finally, we identify a key contribution of CEloff neurons in fear extinction by demonstrating that selective photosilencing of PKCδ CEl neurons during extinction learning prevents extinction memory formation.

PKCδ neurons in the CEl have been demonstrated to play a critical role in the formation of aversive memories by modulating neuronal activity and plasticity in other brain regions^32,33^. Similarly, our study suggests that PKCδ neurons may regulate extinction learning by controlling extinction-related synaptic plasticity locally or downstream of the CEl. Thus, PKCδ neurons may have a more general role in emotional learning by integrating different sensory modalities, valence and attentional signals, thereby flexibly selecting and scaling emotional responses by modulating the activity and plasticity of downstream circuits in motor, autonomic or neuroendocrine centres^12^. Alternatively, distinct subpopulations of PKCδ neurons might control learning in a valence-specific manner.

The exact circuit and plasticity mechanisms by which CEA neuronal activity is altered by extinction remain to be elucidated. A reduction in the activity of (SST-expressing) CElon occurring with extinction could stem from the reversal (depotentiation) of the conditioning-induced strengthening of synaptic inputs from LA and PVT onto CEl SST-expressing neurons^14,18^. Alternatively, extinction may engage additional circuit components that suppress the activity of CElon neurons. These components could be extrinsic to the CEl, such as neurons residing in the amygdala striatal transition area^34^ or ITC clusters^35^, or intrinsic to the CEl itself, for example in the form of local CEl inhibitory circuits. In this context, corticotropin-releasing hormone (CRH)-expressing neurons in the CEl have recently been shown to inhibit local SST-expressing neurons, suppress freezing and regulate extinction^22,24,25^. Of further note, CEl CRH-expressing neurons express an array of other neuropeptides and neuropeptide receptors^36^. Given various neuropeptide systems operate in the CEA to control physiological and behavioural readouts of fear^37,38^, defining novel neuropeptide-expressing CEl subpopulations could help identify novel circuit elements underlying extinction-related decreases in CElon neuron activity.

Previous work has shown that the CElon and CEloff populations can modulate one another’s activity through reciprocal inhibition^15,21,22^. Hence, a reduction in afferent input from CElon neurons following extinction could produce a release of inhibition over the CEloff subpopulation. Interestingly, our finding that optogenetic silencing of PKCδ (CEloff) neurons impairs extinction memory formation, without producing a frank increase in freezing during silencing, indicates that CEloff neurons do not simply gate fear expression but are a locus of plasticity for extinction. Thus, our study indicates that PKCδ/CEloff neuronal activity during CS exposure is required for extinction memory formation.

We infer that the diminution of CS-induced inhibitory responses of CEloff neurons over the course of extinction learning and into retrieval (as shown by our electrophysiological data), and associated inhibition of downstream targets, might be necessary for the induction and/or consolidation of a proper extinction memory—for instance by inhibition of freezing-promoting downstream pathways. Optogenetic inhibition of CEloff neurons during CS exposure over the entire extinction acquisition session prevents this shift in CS responses and could therefore impair downstream plasticity events necessary for the formation of a stable long-term extinction memory. Determining the mechanisms underlying changes in the CS response of CEloff neurons during extinction learning will be another important question for future studies. Notwithstanding, our findings are consistent with a heuristic model in which extinction leads to a reduction in the CS-related activity of CElon neurons and a subsequent disinhibition of the CEloff subpopulation, which, in turn, could suppress CEm output to vlPAG and thereby reduce freezing.

There are a number of caveats to this model. First, it remains possible that extinction alters the regulation of CEloff neurons by upstream inputs other than (or in addition to) CElon neurons. These inputs could include some of the same aforementioned structures known to innervate CElon cells, such as the PVT, amygdala striatal transition area^34^, BLA and ITC clusters. Second, there is compelling evidence that CEm output can be regulated independently of CEl input. CElon neurons can bypass the CEloff→CEm pathway and project directly to vlPAG^18^. Furthermore, extinction is associated with the strengthening of direct, ITC-mediated, feed-forward inhibition of CEm output neurons^39^, in a manner driven by principal cells located in BLA^39^ and infralimbic cortex^40^. The observation that permanent ablation of the entire ITC clusters impairs extinction retrieval further highlights a role for these cells^41^ though, given evidence of significant heterogeneity between individual ITC clusters^28,30^, the precise nature of this role remains unresolved. Nonetheless, it seems likely that ITCs are a key substrate for extinction that may operate in parallel or in concert with CEloff neurons to affect freezing.

Collectively, these prior observations, taken together with the current findings, suggest that while the CEA is an essential node within the broader neural circuitry mediating fear behaviours, the process of extinction likely engages multiple circuit elements that regulate the activity of CEA neurons to modulate freezing. The combination of independent and interacting circuits would endow a system with the dynamic range and flexibility to adjust behavioural responses to fear-related stimuli according to accumulated experience and prevailing environmental conditions. A system for extinction with flexibility and inbuilt redundancy would be of significant adaptive value considering generating an appropriate level of fear behaviour is crucial to survival for many species. In humans, dysfunction of this system, including deficient plasticity in the CEA subpopulations described here, could contribute to the impaired fear extinction reported in patients with anxiety and trauma-related disorders^42,43^.

## Methods

### Animals

Male C57BL6/J mice (B6, Harlan Ltd), 129S1/SvImJ mice (S1, Charles River or Jackson Laboratory) and PKCδ-Cre-CFP mice^21^ were housed by strain (1–2 animals per cage) for 7 days before all experiments, under a 12-h light/dark cycle, and were provided with food and water ad libitum. The ambient temperature in the animal facility was ca. 20 °C and the humidity ca. 30%. In the current study, male mice were used to aid comparability with prior analysis of CEA neurons^15,21^ and in part because heavier male mice were better suited to carrying the electrode implants during locomotion. It will be important and potentially highly informative to modify procedures to enable the study of fear-related CEA neuronal activity in female mice in future work. All animal procedures were executed in accordance with institutional guidelines and were approved by the Veterinary Department of the Canton of Basel-Stadt, the Austrian Animal Experimentation Ethics Board and Austrian Ethical Committees on Animal Care and Use (Bundesministerium für Wissenschaft und Verkehr, Kommission für Tierversuchsangelegenheiten), the National Institute on Alcohol Abuse and Alcoholism Animal Care and the National Institutes of Health guidelines outlined in ‘Using Animals in Intramural Research’.

### Behaviour and optical stimulation

Fear conditioning and extinction took place in two different contexts (context A and B). The conditioning and extinction boxes and the floor were cleaned with 70% ethanol or 1% acetic acid before and after each session, respectively. To score freezing behaviour, an automatic infrared beam detection system placed on the bottom of the experimental chambers (Coulbourn Instruments) was used. The animals were considered to be freezing if no movement was detected for 2 s. On day 1, mice were submitted to a habituation session in context B, in which they received 4 presentations of the CS (total CS duration of 30 s, consisting of 50-ms pips repeated at 0.9 Hz, 2-ms rise and fall; pip frequency: 7.5 kHz or white-noise counterbalanced across animals, 80 dB sound pressure level). Fear conditioning was performed on the same day by pairing the CS with a US (1 s foot shock, 0.6 mA, 5 CS/US pairings; inter-trial interval: 20–180 s). The onset of the US coincided with the offset of the CS. On days 2 and 3, conditioned mice were submitted to extinction training in context B, during which they received 12 presentations of the CS. Retrieval of extinction, spontaneous recovery of conditioned fear (50% freezing cut-off) and context-dependent fear renewal were tested 7 days later in context B and A, respectively, with 4 presentations of the CS^9^. Statistical comparisons were performed with one-way repeated-measures ANOVA followed by Bonferroni post hoc test (*p* < 0.05 was considered significant).

For the quantification of zif268 in PKCδ neurons in the CEl amygdala, mice were submitted to an auditory fear conditioning paradigm in which the CS (total CS duration of 30 s, 10 kHz, 80 dB sound pressure level) was paired to the US (2 s foot shock; 0.6 mA; three CS/US pairings; inter-trial interval: 20–180 s) (TSE operant system). The onset of the US coincided with the offset of the CS. Fear conditioning was always performed in a context (context A) different from that used in the extinction session (context B). Context A was cleaned with water and context B with 70% alcohol followed by water. On the following day, fear memory retrieval and extinction training was performed in context B by presenting 16 CSs with an inter-trial interval of 5 s^44^. The ‘fear expression’ groups received only 2 presentations of the CS following fear conditioning. Freezing behaviour was quantified as an index of fear^45^ in each behavioural session by manually quantifying freezing behaviour; defined as no visible movement except that required for respiration, and converted to a percentage [(duration of freezing within the CS/total time of the CS) × 100] by a trained observer blind to the experimental groups.

For discriminative extinction, mice were habituated on day 1 to 4 presentations of two different CS in context B (total CS duration of 30 s, consisting of 50-ms pips repeated at 0.9 Hz, 2 ms rise and fall; pip frequency: 7.5 kHz or white noise, 80 dB sound pressure level). Both CSs were subsequently paired with a US (1-s foot shock, 0.6 mA, 5 CS/US pairings for each CS; inter-trial interval: 20–180 s). The onset of the US coincided with the offset of the CS. On days 3 and 4, only one of the two CSs was extinguished by 16 and 12 presentations in context B, respectively. At the end of the second extinction session, mice were exposed to 4 presentations of the non-extinguished CS in context B^9^. Statistical comparisons were performed with one-way repeated-measures ANOVA followed by Bonferroni post hoc test (*p* < 0.05 was considered significant).

Optogenetic experiments were performed using a fear conditioning and fear extinction procedure in virally injected PKCδ-Cre positive or negative mice. On day 1, two different CS, CS1 and CS2 (total CS duration of 30 s, consisting of 50-ms pips repeated at 0.9 Hz, 2 ms rise and fall; pip frequency: 7.5 kHz or white noise, 80 dB sound pressure level, counterbalanced across animals) were paired 5 times with the US (1-s foot shock, 0.6 mA, inter-trial interval: 20–180 s). On day 2, fear memory was tested by presenting 4 CS1 and 4 CS2. On day 3, fear extinction was achieved by sequentially presenting 16 CS1 and 16 CS2 (counterbalanced for order across animals). From the 5th to the 16th CS for CS1 and CS2, each CS pip was coupled to light stimulation (−50 ms to +300 ms from pip onset, 20–40 mW) bilaterally delivered through optic fibres (200 µm core diameter, 0.37 NA, Thorlabs GmbH) to the CEl amygdalae.

Optic fibres were connected to a custom-built laser bench using an AOTF (AA Opto-Electronic) to control laser intensity (lasers: MBL473, 473-nm wavelength and MGL593.5, 593.5-nm wavelength, CNILasers). To ensure that animals could move freely, the connecting fibres were suspended over the behavioural context. On day 4, extinction memory was tested by the 4 presentations CS1 and CS2 (counterbalanced for order across animals). Statistical comparisons were performed with two-way repeated-measures ANOVA followed by Bonferroni post hoc test (*p* < 0.05 was considered significant).

### Single-unit recordings and virus injections

Mice were anaesthetized with isoflurane (induction 5%, maintenance 2.5%) in O_2_. Body temperature was maintained with a heating pad (CMA/150, CMA/Microdialysis). Mice were secured in a stereotaxic frame and unilaterally implanted in the amygdala with a multi-wire electrode aimed at the following coordinates: 1.3 mm posterior to bregma; ±2.6 mm lateral to midline; 3.25–3.75-mm deep from the cortical surface. The electrodes consisted of 8–16 individually insulated nichrome wires (13 µm inner diameter, impedance 50–300 kΩ; California Fine Wire) contained in a 26-gauge stainless steel guide cannula. The wires were attached to a 10 pin to 18 pin connector (Omnetics). The implant was secured using cyanoacrylate adhesive gel. After surgery mice were allowed to recover for 7 days. Analgesia was applied before and during the 3 days after surgery (Metacam).

Electrodes were connected to a headstage (Plexon) containing 8–16 unity-gain operational amplifiers. The headstage was connected to a 16-channel computer-controlled preamplifier (gain X-100, band-pass filter from 150 Hz to 9 kHz, Plexon). Neuronal activity was digitized at 40 kHz and band-pass filtered from 250 Hz to 8 kHz, and was isolated by time–amplitude window discrimination and template matching using a Multichannel Acquisition Processor system (Plexon). At the conclusion of the experiment, recording sites were marked with electrolytic lesions before perfusion, and electrode locations were reconstructed with standard histological techniques^15^.

For optical stimulation of PKCδ CEl neurons, PKCδ-Cre+ animals were bilaterally injected into CEl amygdalae with an rAAV serotype 2/7 (Vector Core, University of Pennsylvania), containing a construct conditionally coding for ChR2-2A-eNpHR-2A-Venus^46^ at −1.3 mm posterior and +/− 2.6 mm lateral to bregma at a depth of 3.25–3.75 mm. The use of a 2A-Peptide Self-Processing cassette in the AAV2/7 DIO-EF-1α-ChR2-2A-eNpHR-2A-Venus enables equimolar/isostoichiometric expression of ChR2, eNpHR and Venus in PKCδ neurons to bi-directionally control their activity^42,47^. For identification of the injection site, the virus solution was mixed at 1:1000 with blue fluorescing polymer microspheres (Duke Scientific Corp.). Deeply anesthetized animals were fixed in a stereotactic frame (Kopf Instruments) and the skin above the skull was cut. Glass pipettes (tip diameter 10–20 μm), connected to a Picospritzer III (Parker Hannifin Corporation), were lowered by a Micropositioner (Kopf Instruments) to the depth of 3.75 mm. About 300 nl were pressure injected bilaterally into CEl amygdalae. In the same surgeries 26-gauge stainless steel guide cannulas (Plastics One) were implanted bilaterally along the same track above CEl amygdalae at a depth of −3.25 mm. Guide cannulas were secured using cyanoacrylate adhesive gel (Henkel) and dental cement (Heraeus Dental). To prevent blockage of the cannulas, dummy cannulas (Plastics One) were inserted and fixed. Behavioural experiments were performed after 4 weeks of recovery and expression time and 3 days of handling. After the experiment, optic fibres were removed and animals were perfused for histological analysis of the injection site as described below.

### Single-unit spike sorting and analysis

Single-unit spike sorting was performed using Off-Line Spike Sorter (Plexon) as described^15^. Principal component scores were calculated for unsorted waveforms and plotted on three-dimensional principal component spaces, and clusters containing similar valid waveforms were manually defined. A group of waveforms was considered to be generated from a single neuron if it defined a discrete cluster in principal component space that was distinct from clusters for other units and if it displayed a clear refractory period (>1 ms) in the auto-correlogram histograms. In addition, two parameters were used to quantify the overall separation between identified clusters in a particular channel. These parameters include the J3 statistic, which corresponds to the ratio of between-cluster to within-cluster scatter, and the Davies–Bouldin validity index (DB), which reflects the ratio of the sum of within-cluster scatter to between-cluster separation. High values for the J3 and low values for the DB are indicative of good cluster separation. Control values for this statistic were obtained by artificially defining two clusters from the centred cloud of points in the principal component space from channels in which no units could be detected (Supplementary Fig. 1).

Template waveforms were then calculated for well-separated clusters and stored for further analysis. Clusters of identified neurons were analysed offline for each recording session using principal component analysis and a template-matching algorithm. Only stable clusters of single units recorded over the time course of the entire behavioural training were considered. Long-term single-unit stability isolation was evaluated using Wavetracker (Plexon) in which principal component space-cylinders were calculated from data recorded during behavioural sessions. Straight cylinders suggest that the same set of single units was recorded during the entire training session (Supplementary Fig. 1). We further quantified the similarity of waveform shape by calculating linear correlation (*r*) values between average waveforms obtained over training days (Supplementary Fig. 1). As a control, we computed the *r* values from average waveforms of different neurons.

To avoid analysis of the same neuron recorded on different channels, we computed cross-correlation histograms. If a target neuron presented a peak of activity at a time that the reference neuron fires, only one of the two neurons was considered for further analysis. CS-induced neural activity was calculated by comparing the firing rate after stimulus onset with the firing rate recorded during the 500 ms before stimulus onset (bin size, 50 ms; averaged over blocks of 4 CS presentations consisting of 108 individual sound pips in total) using a *z*-score transformation. *Z*-score values were calculated by subtracting the average baseline firing rate established over the 500 ms preceding stimulus onset from individual raw values and by dividing the difference by the baseline standard deviation. Classification of units was performed by considering a significant *z*-score value within 250 ms after CS onset during the fear test according to the freezing levels. Normalized populations PSTHs were obtained by averaging normalized PSTHs from individual neurons. Statistical comparisons were performed with one-way repeated-measures ANOVA followed by Bonferroni post hoc test or with the Student paired *t*-test for the recall and renewal datasets (*p* < 0.05 was considered significant). Calculations were made in MATLAB and R. Statistical analysis was done in the commercially available software GraphPad Prism and SigmaPlot.

### Optical identification of single units

For optogenetic identification of PKCδ neurons, we used pulses of yellow light (to activate Arch). We used 300-ms pulses, 120 times, with a 2 s inter-pulse interval, at 10 mW light power at the fibre tip. Units were considered as light responsive if they showed significant, time-locked (<10 ms) changes in neuronal activity upon illumination. To determine the onset of inhibition, we used change-point analysis (Change Point Analyzer 2.0, Taylor Enterprises Inc.). As described previously^22,46^, this identifies the time point exhibiting a significant change in neuronal activity relative to the preceding time points. We calculated linear correlation (*r*) values for spontaneous and light-evoked spikes to quantitatively determine the similarity of their waveform shapes.

### Immunohistochemistry and imaging

After completion of experiments, virally injected PKCδ-Cre+ mice were deeply anaesthetized with avertin (0.3 g/kg) Mice were then transcardially perfused with phosphate-buffered saline (PBS) followed by 4% paraformaldehyde (PFA). Coronal, 80-µm-thick brain slices were then cut with a vibratome (VT1000 S, Leica) and stored in PBS containing 0.05% sodium azide. To visualize virus expression, standard immunolabelling procedures were performed on free-floating brain sections: overnight incubation at 4 °C with goat rabbit anti-GFP antibody (1:1000, catalogue no. A11122, Invitrogen), 2 h incubation with anti-rabbit Alexa 488 (1:1000, catalogue no. A11008, Invitrogen). After a final wash, slices were mounted on cover slips and imaged. Mice were included in the analysis if they showed virus expression bilaterally within CEl amygdalae and if fibre tip placement was not more than ~500 µm away from CEl amygdala.

For the quantification of zif268 in PKCδ neurons in the CEl amygdala, mice were killed 2 h after the start of the extinction training session as previously described^30^. Mice were deeply anesthetized using sodium pentobarbitone (200 mg/kg) and transcardially perfused with 20 ml of 0.9% saline followed by 20 ml of 4% paraformaldehyde in phosphate-buffered saline (PBS), pH 7.4. Samples were post-fixed for 2 h in the same fixative at 4 °C and stored in PBS. Coronal sections (40 µm) were cut on a vibratome (Leica Microsystems) and collected in tris buffered saline (TBS). Free-floating sections were incubated in blocking solution (10% BSA and 0.1% Triton X-100 in TBS) then with primary polyclonal rabbit anti-Zif268 antibody (1:2000; Cat. No.: sc-189; Santa Cruz Biotechnology) and monoclonal mouse anti-PKCδ (1:1000, Cat. No.: 610398, BD Transduction Laboratories) for 48 h at 4 °C. The sections were then washed with TBS and incubated for 2 h at room temperature with Cy2-conjugated donkey anti-rabbit (1:500; Cat. No.: 711-225-152; Jackson ImmunoResearch Laboratories) and Alexa Fluor 647-conjugated donkey anti-mouse (1:500; Cat. No.: 717-605-150; Jackson ImmunoResearch Laboratories).

Sections were then attached to microscope slides and coverslipped with FluroGold Antifade reagent. All immunolabelled sections were imaged using an Olympus BX51 microscope equipped with an Olympus XM10 video camera. Images taken under consistent exposure times using a ×20 oil-immersed optical objective lens (UPlanSApo, Olympus Corporation) were digitised and viewed using CellSens Dimension 1.5 software (Olympus Corporation, Tokyo, Japan). The quantification of Zif268 expression in PKCδ positive or negative expressing neurons in the CEl was achieved by manual scoring. Statistical comparisons were performed with a two-way ANOVA followed by Fischer LSD post hoc test (*p* < 0.05 was considered significant).

### Dendritic morphology of CEl neurons

The dendritic morphology of CEl neurons was determined using Golgi stain as described previously^29,48^. Mice were overdosed with xylazine/ketamine and then transcardially perfused with 0.9% saline. Brains were removed and immersed in Golgi-Cox solution (1:1 solution of 5% potassium dichromate and 5% mercuric chloride diluted 4:10 with 5% potassium chromate) for 18 days. Brains were dehydrated, infiltrated with a graded series of celloidins, and embedded in 8% celloidin. Coronal sections were cut at a thickness of 160 μm on a sliding microtome (American Optical 860) and alkalinized, developed, fixed, dehydrated, cleared, mounted, and coverslipped.

Neurons selected for reconstruction did not have truncated branches and were unobscured by neighbouring neurons and glia, with dendrites that were easily discriminable by focusing through the depth of the tissue. In 4–6 sections evenly spaced through the rostral-caudal extent of the CEl amygdala, an average of 4–6 neurons per mouse (average of 2.5 from each hemisphere) were randomly selected (using a random number generator, http://www.randomizer.org) and reconstructed. Neurons were drawn in three-dimensions by an experimenter blind to strain, using a ×100 objective on an Olympus BX41 system microscope using a computer-based neuron tracing system (Neurolucida, MBF Biosciences). The length and number of dendrites, as well as the length and number of terminal branches, was measured for all dendritic arbours. Values were compared between strains using *t*-tests. In addition, to assess the overall amount and location of dendritic material, a three-dimensional version of a Sholl analysis^49^ was performed by measuring the number of dendritic intersections within 10 μm concentric spheres radiating from the soma.

### Statistics and reproducibility

Imaging was repeated independently with similar results in Fig. 5c (*n* = 11 mice) and in Supplementary Fig. 7a (B6 expression, *n* = 8 mice; S1 expression, *n* = 6 mice; B6 extinction, *n* = 8 mice; S1 extinction, *n* = 6 mice). Distinct spike waveforms recorded from different units and sorted using 3D principal component analysis could be observed in all recordings with more than one unit per electrode as shown in Supplementary Fig. 1b.

### Reporting summary

Further information on research design is available in the Nature Research Reporting Summary linked to this article.

## Supplementary information

Supplementary Information

Reporting Summary

## Data Availability

The data that support the findings on this study are available from the corresponding authors upon request. The data that supports the findings of this study are available at https://data.fmi.ch/PublicationSupplementRepo/. Source data are provided with this paper.

## Source data

Source Data

## Code availability

The computer code that supports the findings of this study is available from the corresponding authors upon request.
