# Supplementary material for: Central amygdala micro-circuits mediate fear extinction
Source: Nat Commun. 2021 Jul 6;12:4156. doi: 10.1038/s41467-021-24068-x (PMC8260764; doi:10.1038/s41467-021-24068-x)
Supplement: Supplementary file 1 — Supplementary Information [file 41467_2021_24068_MOESM1_ESM.pdf]

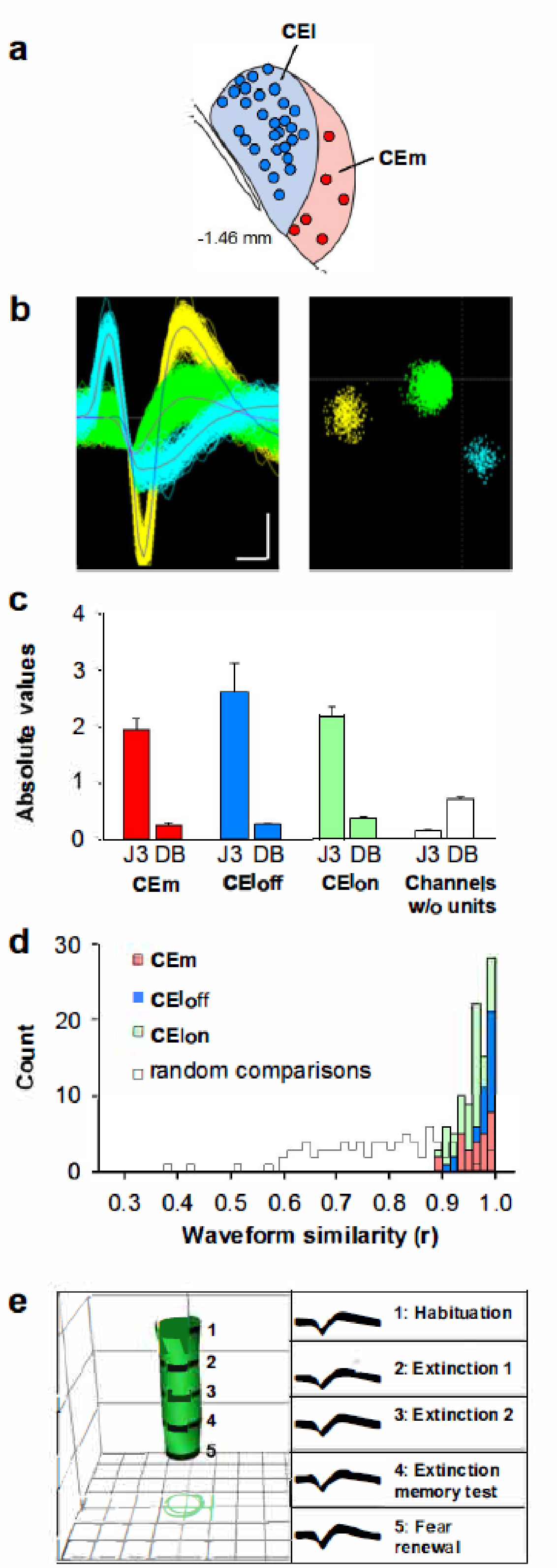


**Fig. S1. Recording sites in CEA and isolation of unit recordings.**

**(a)** Coronal section of the amygdala showing the location of the recording sites of CEl and CEm units. Numbers indicate the antero-posterior coordinates caudal to bregma. **(b)** Left, superimposed waveforms recorded from three different units. Scale bars: 200 µs, 50 µV. Right: Spikes originating from individual units were sorted using 3D principal component analysis. **(c)** Quantitative J3 and Davies Bouldin validity index (DB) statistics calculated for CEm (*n* = 27), CEloff (*n* = 48) and CElon (*n* = 98) neurons. Control values were obtained using two clusters defined from the centred cloud of points from channels in which no units could be detected. High values for the J3 and low values for the DB are indicative of good single-unit isolation. All values are expressed as means ± SEM. **(d)** In addition, to quantitatively evaluate similarity of different spike shapes recorded on different days, linear correlation values between time-shifted average waveforms were calculated for CEm, CEloff and CElon neurons. As a control we computed the r values from average waveforms of different neurons (random comparisons). The maximum r value across time shifts was used to quantify similarity (r = 1 indicates identical spike shapes). **(e)** Left, stability of clustered waveforms during long-term recordings was assessed by calculating principal component (PC) space cylinders. Straight cylinders suggest that the same set of single units was recorded during the entire training session. Right, superimposed waveforms used to calculate the PC space cylinder recorded during habituation, at the beginning of the first extinction, at the end of the second extinction, extinction memory and fear renewal. Source data are provided as a Source Data file.

Figure 1

| Experiment | Habituation | FC | Extinction 1 | Extinction 2 |
|---|---|---|---|---|
| Day | 1 | 1 | 2 | 3 |
| Context | A (safe) | B (FC) | A (safe) | A (safe) |
| CS | 4 | 5 | 12 | 12 |
| US | . | 5 | . | . |

Figure 2

| Experiment | Habituation | FC | Extinction 1 | Extinction 2 |
|---|---|---|---|---|
| Day | 1 | 1 | 2 | 3 |
| Context | A (safe) | B (FC) | A (safe) | A (safe) |
| CS1 | 4 | 5 | 16 | 12 |
| US1 | . | 5 | . | . |
| CS2 | 4 | 5 | . | 4 |
| US2 | . | 5 | . | . |

Figure 3

| Experiment | Habituation | FC | Ext. 1 | Ext. 2 | Ext. memory test | Fear renewal |
|---|---|---|---|---|---|---|
| Day | 1 | 1 | 2 | 3 | 10 | 10 |
| Context | A (safe) | B (FC) | A (safe) | A (safe) | A (safe) | B (FC) |
| CS | 4 | 5 | 12 | 12 | 4 | 4 |
| US | . | 5 | . | . | . | . |

Figure 4

| Experiment | Habituation | FC | Extinction 1 | Extinction 2 |
|---|---|---|---|---|
| Day | 1 | 1 | 2 | 3 |
| Context | A (safe) | B (FC) | A (safe) | A (safe) |
| CS | 4 | 5 | 12 | 12 |
| US | . | 5 | . | . |

Figure 5

| Experiment | FC | Fear memory test | Ext. learning | Ext. memory test |
|---|---|---|---|---|
| Day | 1 | 2 | 3 | 4 |
| Context | B (FC) | A (safe) | A (safe) | A (safe) |
| CS1 | 5 | 4 | 16 | 4 |
| US1 | 5 | . | . | . |
| CS2 | 5 | 4 | 16 | 4 |
| US2 | 5 | . | . | . |

**Fig. S2. Behavioural protocols.**

Experimental designs associated with Figs 1-5. FC: fear conditioning. CS: conditioned stimuli. US: unconditioned stimuli. Ext: extinction.

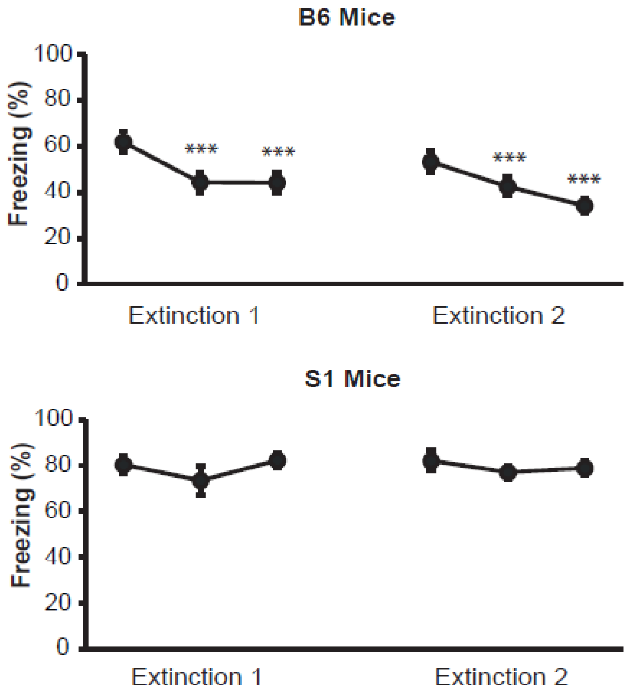


**Fig. S3. B6 mice exhibit normal fear extinction whereas S1 mice are impaired.**

**Top**, Summary plots illustrating behavioural data. During fear extinction, B6 mice exhibited a decrease in freezing responses to CS exposure. B6 mice: $n = 27$; freezing, blocks of 4CSs, extinction 1, CS1-4: 61.9 ± 4.6%, CS5-8: 44.3 ± 4.6%, CS9-12: 44.1 ± 4.4%, extinction 2, CS1-4: 53.2 ± 4.7%, CS5-8: 42.5 ± 4.1%, CS9-12: 34.1 ± 3.4%. One-way repeated measures ANOVA $F_{(5,130)} = 12.3$, $p < 0.001$. The second and third CS blocks of extinction 1 and extinction 2 show a decrease in freezing levels (post-hoc Bonferroni *t*-test vs. first CS block of extinction 1, $p < 0.001$). **Bottom**, during fear extinction, S1 mice did not decrease their freezing responses to CS exposure. S1 mice: $n = 7$; freezing, blocks of 4CSs, extinction 1, CS1-4: 80.3 ± 3.8%, CS5-8: 73.4 ± 6.1%, CS9-12: 82.2 ± 3.2%, extinction 2, CS1-4: 82.0 ± 4.4%, CS5-8: 77.0 ± 3.0%, CS9-12: 78.9 ± 3.2%. One-way repeated measures ANOVA $F_{(5,30)} = 0.74$, $p = 0.599$. All values are expressed as means ± SEM. Source data are provided as a Source Data file.

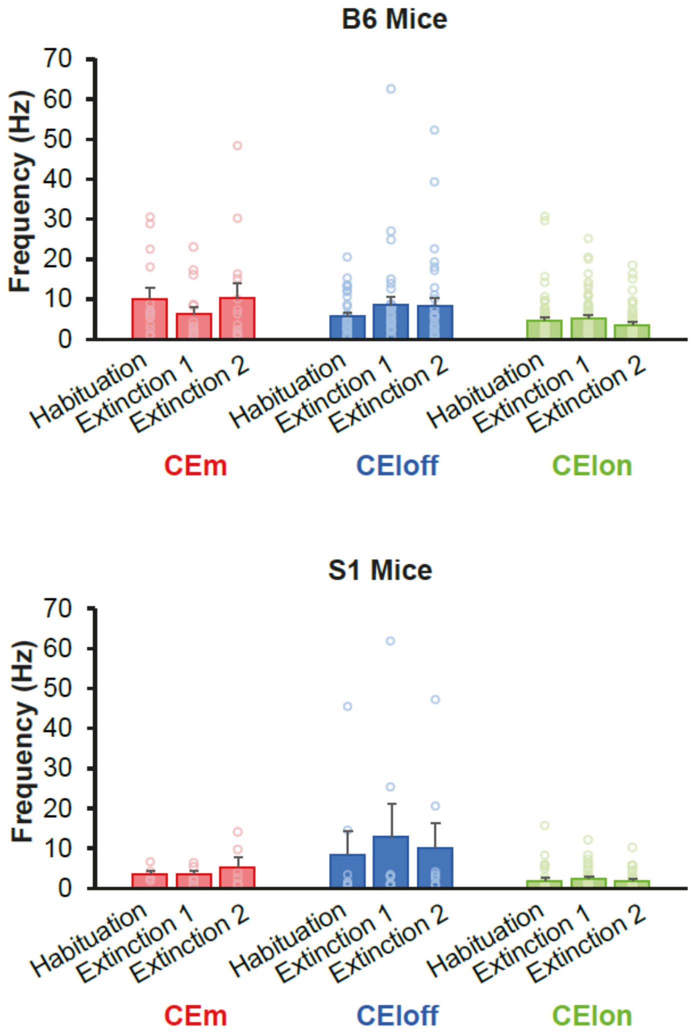


**Fig. S4. Spontaneous activity of CEm, CEloff, CElon neurons in B6 and S1 mice during fear extinction.**

Although fluctuations in spontaneous activity were observed in individual neurons, no significant changes in spontaneous activity could be observed at the population level for CEm, CEloff and CElon neurons in B6 (top) or S1 mice (bottom) during habituation, extinction 1 and extinction 2 (one-way repeated measures ANOVA $p > 0.05$ for each CEA population). Bar plots are expressed as means ± SEM. Circles are firing rate values of individual neurons (calculated as the mean firing rate activity measured 500 ms before pips, over a total of 108 pips corresponding to 4 CSs). Source Data are provided as a Source Data file.

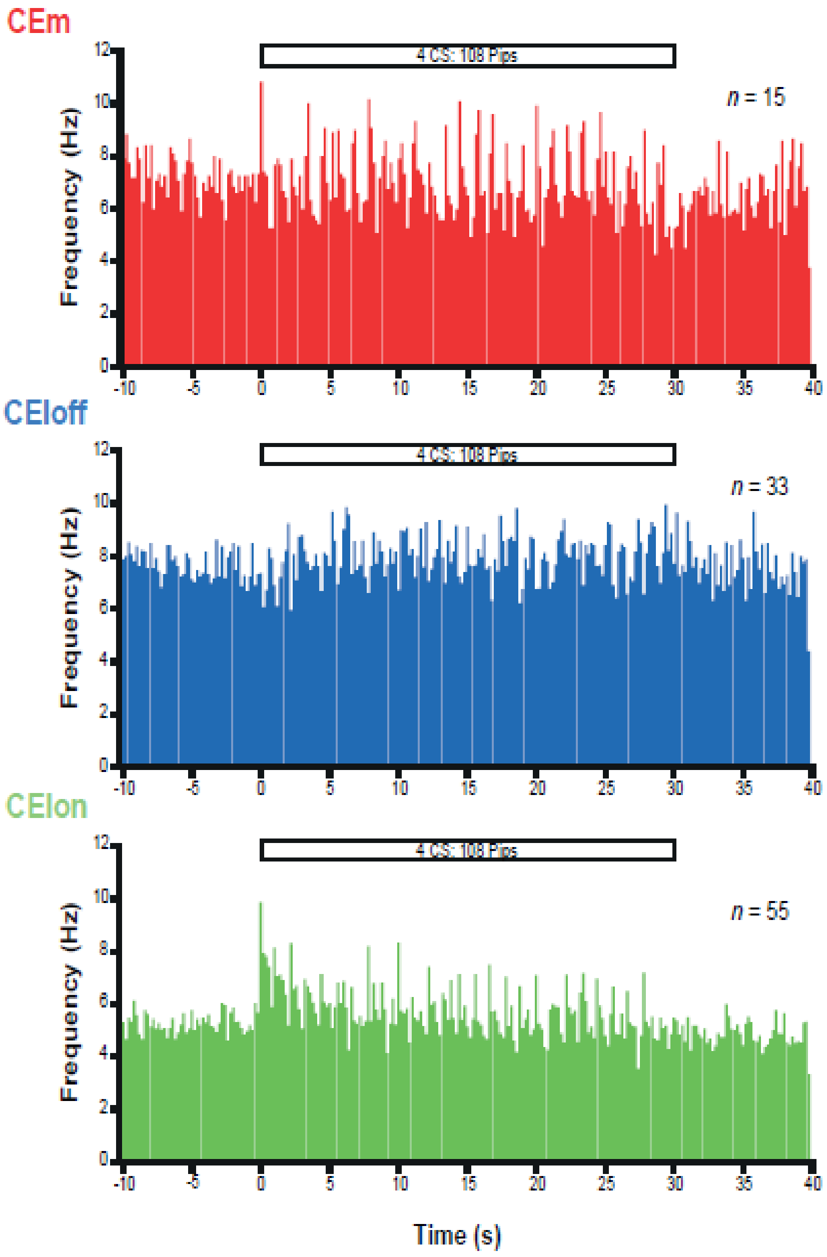


**Fig. S5. Time-course of neuronal activity over the entire CS.**

Time histograms illustrate absolute firing rates averaged across the first four CS presentations of extinction 1 for CEm (*n* = 15), CEloff (*n* = 33), CElon (*n* = 55) neurons. Phasic responses can be seen upon exposure to auditory pips. However, no overall tonic changes in firing rate was observed. Source data are provided as a Source Data file.

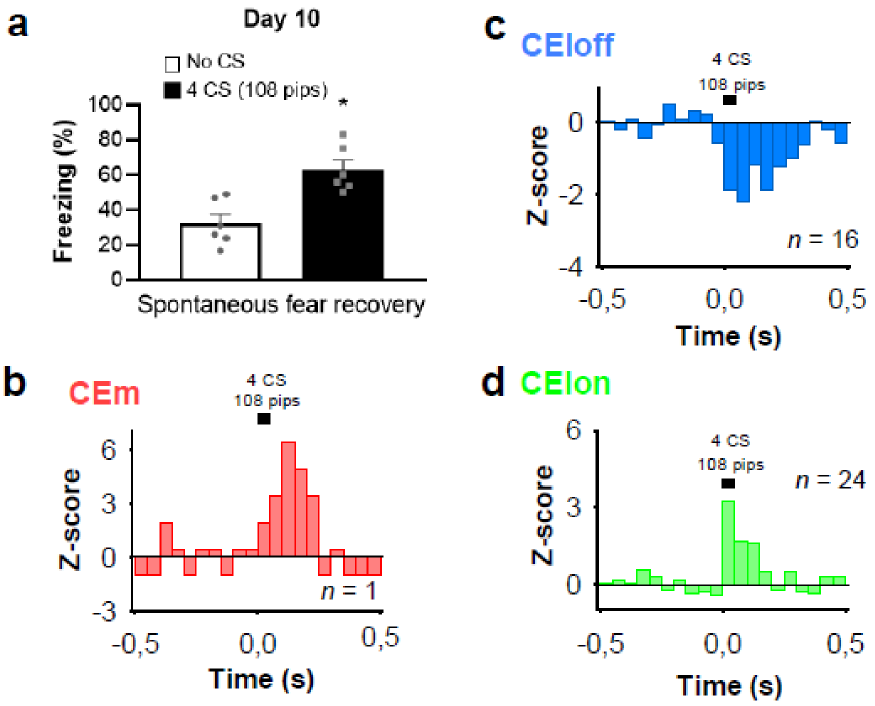


**Fig. S6. Spontaneous recovery of conditioned fear is associated with a recovery of CS-induced activity in subpopulations of CEA neurons.**

(**a**) Summary plots illustrating behavioural data. Seven days after extinction learning, a subset of B6 mice exhibited spontaneous recovery of conditioned fear responses when tested in the same context in which extinction training took place. B6 mice: $n$ = 6; freezing, spontaneous recovery, no CS: 32.9 ± 5.3%, CS: 63.5 ± 5.3%, blocks of 4 CSs, paired t-test, two-sided, $p$ = 0.027. Bar plots are expressed as means ± SEM. (**b**) One CEm neuron exhibited an increase in CS-related firing during spontaneous fear recovery as shown in the normalized peri-stimulus time histogram. (**c**) CEloff neurons ($n$ = 16) displayed a CS-related decrease in firing during spontaneous fear recovery as shown in normalized and averaged population peri-stimulus time histograms. (**d**) CElon neurons ($n$ = 24) exhibited an increase in CS-related firing during spontaneous fear recovery as shown in the normalized and averaged population peri-stimulus time histograms. All individual neurons of each CEA population had significant z-score values upon CS presentation (4 CSs during spontaneous fear recovery). The duration of the auditory stimulus is indicated (black bar; CS: 50 ms). Source data are provided as a Source Data file.

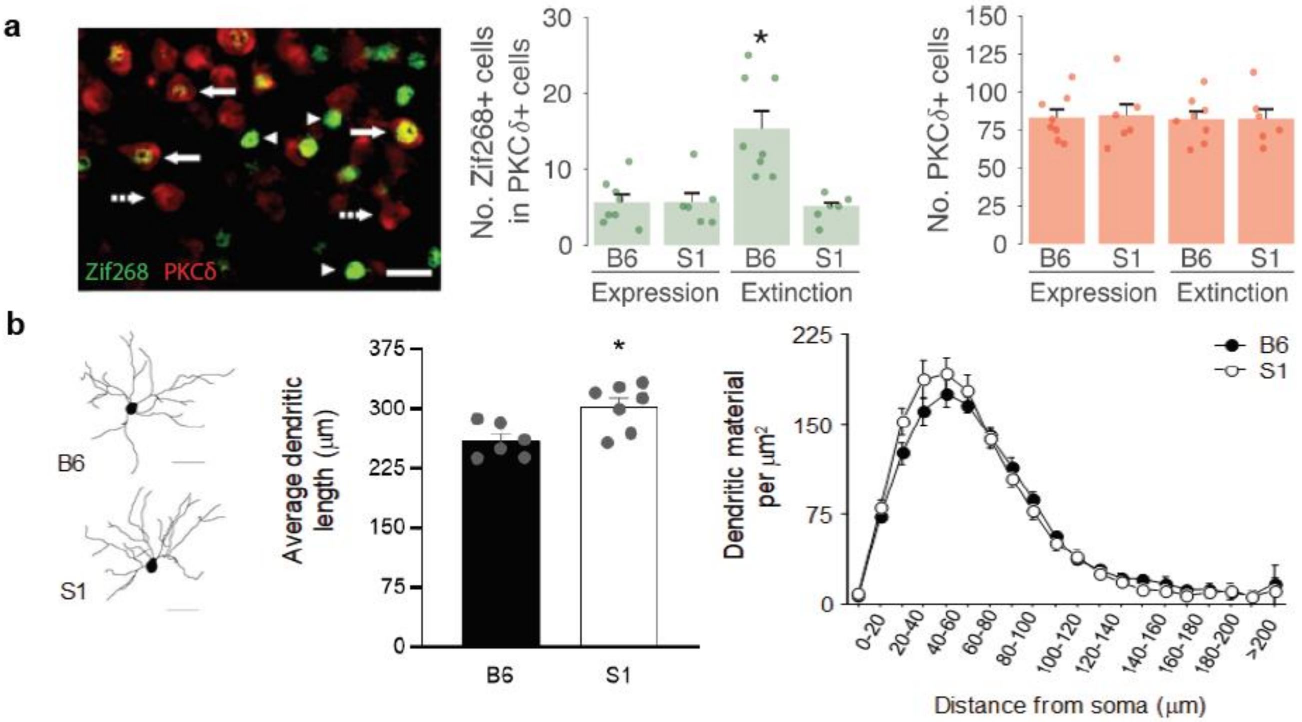


**Fig. S7. Impaired extinction correlates with differential IEG expression and dendritic structure in different subpopulations of CEA neurons.**

**(a)** Left, representative image from a B6 mouse subjected to fear extinction illustrating Zif268 expression in PKCδ nuclei (double labelling in yellow, solid white arrows), Zif268 expression in PKCδ$^{-}$ nuclei (single labelling in green, white arrow heads) and PKCδ CEl neurons not expressing Zif268 (single labelling in red, white dashed arrows). Scale bar: 10 µm. Middle**,** successful fear extinction in B6 mice enhanced the percentage of PKCδ/Zif268 co-labelled cells as compared to S1 mice impaired in fear extinction: ANOVA $F_{(1,25)} = 8.34$, $p = 0.008$ followed by post-hoc Fischer's LSD test between the number of co-labelled cells B6 mice during extinction versus fear expression. Neither fear memory expression in B6 and S1 mice, nor impaired fear extinction in S1 mice increased Zif268 expression in PKCδ nuclei. These results reveal selective extinction-related induction of Zif268 expression in PKCδ CEl nuclei in B6 mice compared to S1 mice. Statistical test is two-sided. Right, no difference in the number of PKCδ cells was observed between behavioural sessions or B6 and S1 mice: ANOVA $F_{(1,25)} = 0.06$, $p = 0.88$. Statistical test is two-sided. **(b)** Left, examples of reconstructed CEl neurons in B6 and S1 mice. Middle, dendrites in CEl neurons were on average longer in S1 compared to B6 mice (303 ± 11µm versus 260 ± 9% µm, p = 0.011 t-test, two-sided). Right, Sholl analysis showing the distribution of dendritic structures, in relation to distance from the soma, in S1 ($n = 7$) and B6 mice ($n = 6$), $n$ = 4-6 neurons per mouse. All values are expressed as means ± SEM. Source data are provided as a Source Data file.

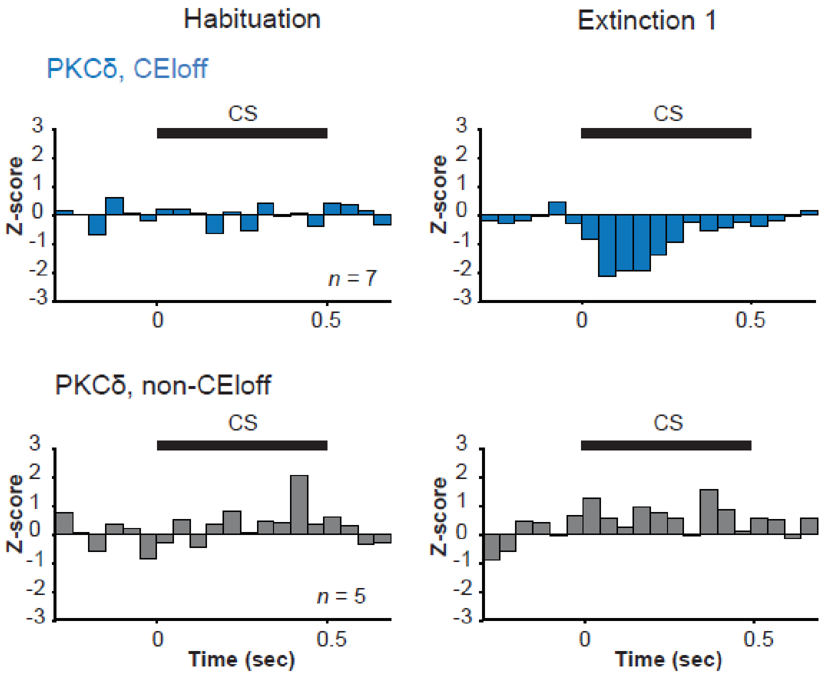


**Fig. S8. Fear conditioning-induced activity in CEl/PKCδ neurons.**

**Top**, a subset of PKCδ neurons (7/12) exhibited an inhibitory response to the CS at the beginning of the first extinction session and thus were classified as CEloff neurons; the inhibitory response was absent during habituation. **Bottom**, the remainder of the PKCδ neurons (5/12) exhibited little CS-induced activity during habituation and at the beginning of the first extinction session. Source data are provided as a Source Data file.

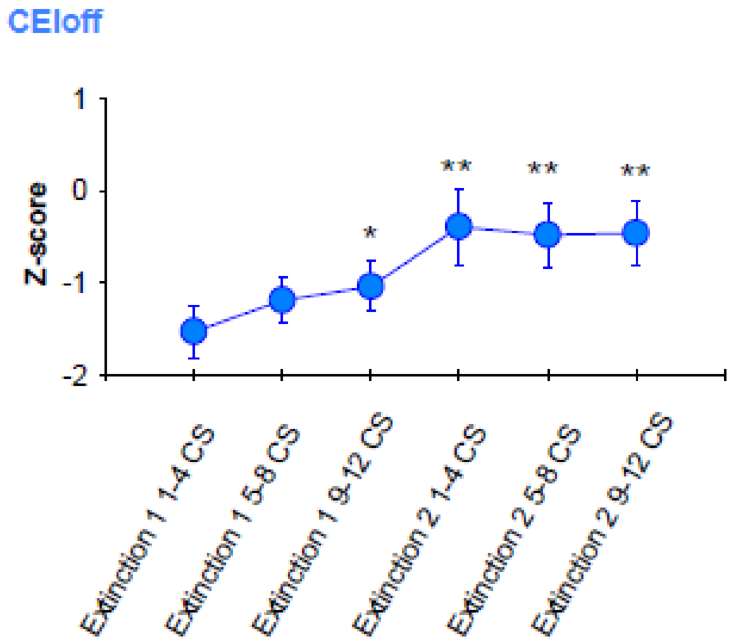


**Fig. S9. Time-course of phasic CS-induced inhibition of CEloff neurons during fear extinction.**

CEloff neurons exhibited a CS-related decrease in firing at the beginning of the first extinction session (extinction 1) followed by a gradual reduction of this inhibitory response over the two extinction sessions. CEloff neurons: $n$ = 33 units from 18 mice; $z$-score, extinction 1, CS1-4: -1.53 ± 0.28, CS5-8: -1.19 ± 0.25, CS9-12: -1.04 ± 0.27, extinction 2, CS1-4: -0.39 ± 0.41, CS5-8: -0.48 ± 0.35, CS9-12: -0.46 ± 0.34. One-way repeated measures ANOVA on ranks, $\chi 2(5,28) = 22.0$, $p < 0.001$. The third CS block of extinction 1 and all CS blocks of extinction 2 showed a reduction of the CS-induced phasic inhibition (post-hoc Dunn's test vs. first CS block of extinction 1, *$p < 0.05$, **$p < 0.01$). All individual CEloff neurons had significant $z$-score values upon CS presentation (first 4 CSs during extinction 1). Changes in $z$-score values are quantified by comparing mean z-score values after pip onset. All values are expressed as means ± SEM. Source data are provided as a Source Data file.

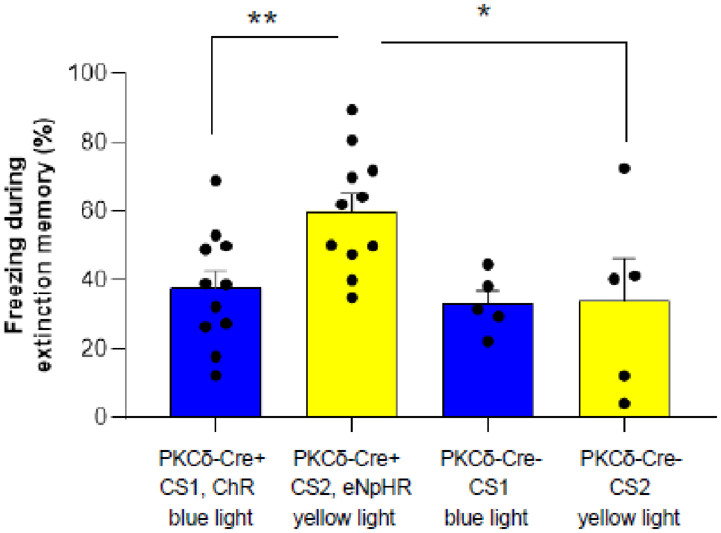


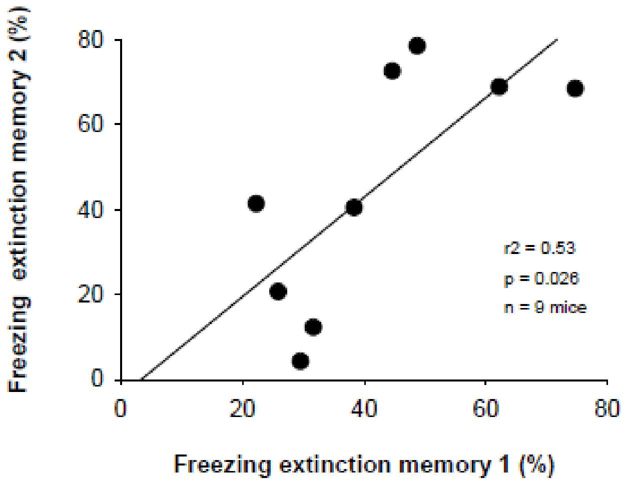


**Figure S10. Optogenetic inhibition of PKCδ/CEloff neurons reduces extinction memory.**

Top, freezing values during extinction memory test upon optogenetic manipulations or in control experiments (related to Fig. 5). PKCδ-Cre+/CS1/ChR2/blue light category (B6 mice: n = 11); freezing, extinction memory, CS1: 37.7 ± 5.0%. PKCδ-Cre+/CS2/eNpHR/yellow light category (B6 mice: n = 11), extinction memory, CS1: 60.1 ± 5.2%. PKCδ-Cre-/CS1/blue light category (B6 mice: n = 5), extinction memory, CS1: 33.2 ± 3.8%. PKCδ-Cre-/CS2/yellow light category (B6 mice: n = 5), extinction memory, CS1: 34.1 ± 12.1%, (blocks of 4CSs). There was a significant main effect of mouse genotype on freezing levels during fear extinction memory tests (two-way ANOVA $F(1,28)= 4.97$, $p = 0.034$). The PKCδ-Cre+/CS2/eNpHR/yellow light group exhibited higher freezing levels (lower extinction memory compared to the PKCδ-Cre-/CS1/ChR2/blue light group upon CS presentations during extinction memory (post-hoc Bonferroni t-test, $p = 0.007$) as well as compared to the PKCδ-Cre-/CS2/yellow light group (post-hoc Bonferroni t-test, $p = 0.012$). All values are expressed as means ± SEM. Bottom, freezing values during the fear extinction memory test for two extinguished CSs significantly correlate in a group of control mice (Pearson correlation coefficient: $r^2 = 0.53$, n = 9 mice, $p = 0.026$, two-sided; see protocol Fig. 5f). Source data are provided as a Source Data file.

| number of recorded neurons | non-responding neurons | CEm responding neurons | CEloff neurons | CElon neurons | B6 mice |
|---|---|---|---|---|---|
| 6 | 3 | | 3 | | 1 |
| 4 | 1 | | | 3 | 2 |
| 7 | 4 | | 3 | | 3 |
| 2 | | | 1 | 1 | 4 |
| 4 | | | 2 | 2 | 5 |
| 1 | | | 1 | | 6 |
| 7 | 4 | | | 3 | 7 |
| 3 | | | | 3 | 8 |
| 8 | 5 | | | 3 | 9 |
| 13 | 1 | 6 | 4 | 2 | 10 |
| 1 | | 1 | | | 11 |
| 3 | | 3 | | | 12 |
| 6 | 5 | | 1 | | 13 |
| 6 | 3 | | 2 | 1 | 14 |
| 5 | 1 | 4 | | | 15 |
| 6 | 1 | | 2 | 3 | 16 |
| 9 | | | 4 | 5 | 17 |
| 10 | 4 | | | 6 | 18 |
| 6 | 1 | | 1 | 4 | 19 |
| 7 | 1 | | 2 | 4 | 20 |
| 9 | 3 | | 1 | 5 | 21 |
| 3 | 2 | | 1 | | 22 |
| 9 | 7 | | 1 | 1 | 23 |
| 4 | 1 | | 1 | 2 | 24 |
| 17 | 11 | | 2 | 4 | 25 |
| 1 | | 1 | | | 26 |
| 4 | | | 1 | 3 | 27 |
| **161** | **58** | **15** | **33** | **55** | **total number of neurons** |

**Table 1. Number of recorded neurons per category (non-responsive neurons, CEm responding units, CEloff neurons, CElon neurons) and per mice (related to Figure 1).**

| number of recorded neurons | non-responding neurons | CEm responding neurons | CElоff neurons | CElon neurons | B6 mice |
|---|---|---|---|---|---|
| 1 | | 1 | | | 1 |
| 6 | 2 | 4 | | | 2 |
| 7 | 3 | | 1 | 3 | 3 |
| 2 | 1 | | 1 | | 4 |
| 2 | | | 2 | | 5 |
| 4 | 1 | | | 3 | 6 |
| 6 | 2 | | 2 | 2 | 7 |
| 4 | 1 | | | 3 | 8 |
| 1 | | 1 | | | 9 |
| 3 | 1 | | 1 | 1 | 10 |
| **36** | **11** | **6** | **7** | **12** | **total number of neurons** |

**Table 2. Number of recorded neurons per category (non-responsive neurons, CEm responding units, CEloff neurons, CElon neurons) and per mice (related to Figure 2).**

| **number of recorded neurons** | **non-responding neurons** | **CEm responding neurons** | **CEloff neurons** | **CElon neurons** | **B6 mice** |
|---|---|---|---|---|---|
| 4 | | 4 | | | 1 |
| 22 | 11 | | 6 | 5 | 2 |
| 2 | 1 | | 1 | | 3 |
| 5 | | | | 5 | 4 |
| **33** | **12** | **4** | **7** | **10** | **total number of neurons** |

**Table 3. Number of recorded neurons per category (non-responsive neurons, CEm responding units, CEloff neurons, CElon neurons) and per mice (related to Figure 3).**

| **number of recorded neurons** | **non-responding neurons** | **CEm responding neurons** | **CEloff neurons** | **CElon neurons** | **S1 mice** |
|---|---|---|---|---|---|
| 10 | 4 | | 1 | 5 | 1 |
| 3 | 1 | | 1 | 1 | 2 |
| 13 | 5 | | 1 | 7 | 3 |
| 21 | 4 | 4 | 2 | 11 | 4 |
| 11 | 4 | | 2 | 5 | 5 |
| 7 | 5 | 2 | | | 6 |
| 7 | 4 | | 1 | 2 | 7 |
| **72** | **27** | **6** | **8** | **31** | **total number of neurons** |

**Table 4. Number of recorded neurons per category (non-responsive neurons, CEm responding units, CEloff neurons, CElon neurons) and per mice (related to Figure 4).**

| | Return<br>to extinction context | Return to fear conditioning context |
|---|---|---|
| Mice | **Extinction memory**<br>**Low fear**<br>**CS freezing** | **Fear renewal**<br>**High fear**<br>**CS freezing** |
| Mouse #1 | 14.8 % | 77.6 % |
| Mouse #2 | 45.3 % | 91.6 % |
| Mouse #3 | 37.0 % | 58.7 % |
| Mouse #4 | 33.0 % | 64.3 % |
| | **Fear memory**<br>**High fear**<br>**CS freezing** | **Fear memory**<br>**High fear**<br>**CS freezing** |
| Mouse #5 | 83.6 % | 58.7 % |
| Mouse #6 | 60.9 % | 77.6 % |
| Mouse #7 | 75.2 % | 91.6 % |
| Mouse #8 | 54.8 % | 58.7 % |
| Mouse #9 | 50.4 % | 63.8 % |
| Mouse #10 | 56.3 % | 76.7 % |

**Table 5. Freezing levels during long-term memory tests.**
