# Supplementary material for: Central amygdala micro-circuits mediate fear extinction
Source: Nat Commun. 2021 Jul 6;12:4156. doi: 10.1038/s41467-021-24068-x (PMC8260764; doi:10.1038/s41467-021-24068-x)
Supplement: Supplementary file 2 — Reporting Summary [file 41467_2021_24068_MOESM2_ESM.pdf]

nature research

Corresponding author(s): Stéphane Ciocchi, Andreas Lüthi

Last updated by author(s): Mar 11, 2021

Reporting Summary

Nature Research wishes to improve the reproducibility of the work that we publish. This form provides structure for consistency and transparency in reporting. For further information on Nature Research policies, see our Editorial Policies and the Editorial Policy Checklist.

Statistics

For all statistical analyses, confirm that the following items are present in the figure legend, table legend, main text, or Methods section.

n/a	Confirmed	
☐	☒	The exact sample size (*n*) for each experimental group/condition, given as a discrete number and unit of measurement
☐	☒	A statement on whether measurements were taken from distinct samples or whether the same sample was measured repeatedly
☐	☒	The statistical test(s) used AND whether they are one- or two-sided<br>*Only common tests should be described solely by name; describe more complex techniques in the Methods section.*
☒	☐	A description of all covariates tested
☐	☒	A description of any assumptions or corrections, such as tests of normality and adjustment for multiple comparisons
☐	☒	A full description of the statistical parameters including central tendency (e.g. means) or other basic estimates (e.g. regression coefficient) AND variation (e.g. standard deviation) or associated estimates of uncertainty (e.g. confidence intervals)
☐	☒	For null hypothesis testing, the test statistic (e.g. *F*, *t*, *r*) with confidence intervals, effect sizes, degrees of freedom and *P* value noted<br>*Give P values as exact values whenever suitable.*
☒	☐	For Bayesian analysis, information on the choice of priors and Markov chain Monte Carlo settings
☒	☐	For hierarchical and complex designs, identification of the appropriate level for tests and full reporting of outcomes
☐	☒	Estimates of effect sizes (e.g. Cohen's *d*, Pearson's *r*), indicating how they were calculated

Our web collection on statistics for biologists contains articles on many of the points above.

Software and code

Policy information about availability of computer code

Data collection	Plexon v3.3.3, Coulbourn Instruments, Olympus BX51 microscope ,Neurolucida (MBF Biosciences)
Data analysis	Matlab 2020, R 2010, GraphPad Prism 8.4.3, Sigma Stats 2014, WaveTracker (Plexon Inc., v3.3.3), CellSens Dimension 1.5, Change Point Analyzer 2.0 (Taylor Enterprises Inc.),

For manuscripts utilizing custom algorithms or software that are central to the research but not yet described in published literature, software must be made available to editors and reviewers. We strongly encourage code deposition in a community repository (e.g. GitHub). See the Nature Research guidelines for submitting code & software for further information.

Data

Policy information about availability of data

All manuscripts must include a data availability statement. This statement should provide the following information, where applicable:

- Accession codes, unique identifiers, or web links for publicly available datasets
- A list of figures that have associated raw data
- A description of any restrictions on data availability

The data that support the findings on this study are available from the corresponding authors upon request.

Field-specific reporting

Life sciences study design

All studies must disclose on these points even when the disclosure is negative.

Sample size	We have estimated the sample size required for each group based on a statistical power of 80% with a p value < 0.05 using G*Power software, based on an expected effect size of 1 (estimated from previous experimental data), and added an allowance based on the success rate of these experiments.
Data exclusions	no data exclusion
Replication	The exact number of repetitions (individual data points from distinct neurons or animals) are indicated in figures, legends and supplementary tables. Results described throughout the paper were reproduced. Multiple rounds of experimentation were required, i.e., from multiple mice, which were averaged for the presented datasets. Data were acquired from mice from multiple litters. No issues were identified in reproducing any of the reported findings within groups. However, we did not use replication per se (as in multiple separate cohorts of several subjects). All protocols, reagents and mouse lines used for the experiments are described in detail in the Methods section, and further information can be requested from the corresponding authors to ensure that our findings can be replicated in other laboratories.
Randomization	Random selection of neurons to be reconstructed (using a random number generator, http://www.randomizer.org/); random selection of mice to group allocation
Blinding	The analysis of behaviour and optogenetic experiments were automatically performed using custom-made routines. Animals were handled similarly during experiments and in the animal facility which led us to an experimental design without blinding.

Reporting for specific materials, systems and methods

We require information from authors about some types of materials, experimental systems and methods used in many studies. Here, indicate whether each material, system or method listed is relevant to your study. If you are not sure if a list item applies to your research, read the appropriate section before selecting a response.

Materials & experimental systems

n/a	Involved in the study	
☐	☒	Antibodies
☒	☐	Eukaryotic cell lines
☒	☐	Palaeontology and archaeology
☐	☒	Animals and other organisms
☒	☐	Human research participants
☒	☐	Clinical data
☒	☐	Dual use research of concern

Methods

n/a	Involved in the study	
☒	☐	ChIP-seq
☒	☐	Flow cytometry
☒	☐	MRI-based neuroimaging

Antibodies

Antibodies used	Rabbit anti-GFP antibody; Zif268 antibody; monoclonal mouse anti-PKCδ; Cy2 – conjugated donkey anti-rabbit and Alexa Fluor 647 – conjugated donkey anti-mouse; Goat anti-rabbit Alexa 488.
Validation	Rabbit anti-GFP antibody (1:1,000, catalogue no. A11122, Invitrogen); Zif268 antibody (1:2000; Cat. No.: sc-189; Santa Cruz Biotechnology); monoclonal mouse anti-PKCδ (1:1000, Cat. No.: 610398, BD Transduction Laboratories); Cy2 – conjugated donkey anti-rabbit (1:500; Cat. No.: 711-225-152; Jackson ImmunoResearch Laboratories) and Alexa Fluor 647 –conjugated donkey anti-mouse (1:500; Cat. No.: 717-605-150; Jackson ImmunoResearch Laboratories); Goat anti-rabbit Alexa 488 (1:1000, catalogue no. A11008, Invitrogen). All antibodies are widely used commercially available antibodies. Antibodies were validated in WB and IHC by the manufacturer or by previously published studies. Specificity and suitability of secondary antibodies was further validated by omitting preceding primary antibody incubation.

Animals and other organisms

Policy information about studies involving animals; ARRIVE guidelines recommended for reporting animal research

Laboratory animals	Male C57BL6/J mice (B6, Harlan Ltd), 129S1/SvImJ mice (S1, Charles River or Jackson Laboratory) and PKCδ-Cre-CFP mice (Haubensak et al., 2010); male mice; 2-8 months old. Mice were housed by strain (1 -2 animals per cage) for 7 days before all experiments, under a 12 h light/dark cycle, and were provided with food and water ad libitum. The ambient temperature in the animal facility was ca. 20 degrees Celsius and the humidity ca. 30%.
Wild animals	no wild animals were used in the study
Field-collected samples	No field collected samples were used in the study

Ethics oversight	Veterinary Department of the Canton of Basel-Stadt, the Austrian Animal Experimentation Ethics Board and Austrian Ethical Committees on Animal Care and Use (Bundesministerium für Wissenschaft und Verkehr, Kommission für Tierversuchsangelegenheiten), the National Institute on Alcohol Abuse and Alcoholism Animal Care and the National Institutes of Health guidelines outlined in 'Using Animals in Intramural Research'.

Note that full information on the approval of the study protocol must also be provided in the manuscript.
